# Rural Community Toolbox to Help Battle Opioid Epidemic

**DOI:** 10.13023/jah.0204.01

**Published:** 2020-09-01

**Authors:** Liz Carey

**Affiliations:** The Daily Yonder

**Keywords:** Appalachia, rural health, opioid epidemic, drug use

## Abstract

An online data repository and toolbox created by the Office of National Drug Control Policy is designed to provide communities with knowledge and resources to fight the opioid epidemic according to their unique needs.

An online data repository and toolbox created by the Office of National Drug Control Policy are designed to provide communities with knowledge and resources to fight the opioid epidemic according to their unique needs.

The website connects rural leaders to 16 different federal agencies, providing those with substance use disorder or their families a way to find treatment. It also provides rural leaders with a Rural Community Action Guide and Rural Community Toolbox Fact Sheet (see Additional Files) to help educate leaders on steps to take to combat opioid addiction and other drug crises in their communities.

“Research shows that people living in rural America who need help are falling through the cracks, often losing their lives. Treatment services are insufficient to meet rural demand. Access to quality medical care, resources, and training is limited in rural communities, particularly for specialized populations, such as pregnant women, parents, and seniors,” the Rural Community Action Guide said.

“Further, drug courts, which are known to be significantly more effective than incarceration for non-violent offenders, are not available in many rural areas.”

According to the guide, between 1999 and 2015, drug overdose deaths in rural counties jumped by 325%, compared to 198% in metropolitan areas. In 2017, the Guide said, the American Farm Bureau Federation and National Farmers Union conducted a survey that found nearly 50% of rural Americans, and 74% of farmers have been directly affected by opioid misuse. A 2019 Harvard University study released a survey of rural Americans that identified drug addiction as the biggest challenge facing rural communities.

The online toolbox also includes a Community Assessment Tool that will allow communities to search county-level data online to see what is happening in their county. The interactive resource will provide them with information on things like drug overdose deaths (http://overdosemappingtool.norc.org/) and socioeconomic factors that tend to drive substance use, as well as layers of information on things like broadband availability, transportation, treatment facilities, healthcare professional shortages, economic development districts, and persistent poverty.

“We are equipping these communities with resources like the Rural Community Toolbox to help them continue the fight and connect Americans to evidence-based prevention and treatment services,” a spokesperson for the U.S. Department of Health and Human Services said.

The toolbox is a result of the Federal Rural Interagency Working Group on Substance Use Disorder, established in July 2018. The Working Group developed the Federal Rural Resources Guide for rural leaders that provided a comprehensive listing of the federal programs rural leaders could use in combating opioid use disorder. Released in October 2018, it became the foundation of the toolbox.

## Supplementary Information





