# Supplementary material for: Rural Community Toolbox to Help Battle Opioid Epidemic
Source: J Appalach Health. 2020 Sep 1;2(4):1–3. doi: 10.13023/jah.0204.01 (PMC9150490; doi:10.13023/jah.0204.01)
Supplement: Supplementary file 1 [file 2.4.1CareyAdditionalFile#1.pdf]

Rural Community Action Guide

Building Stronger, Healthy, Drug-Free Rural Communities

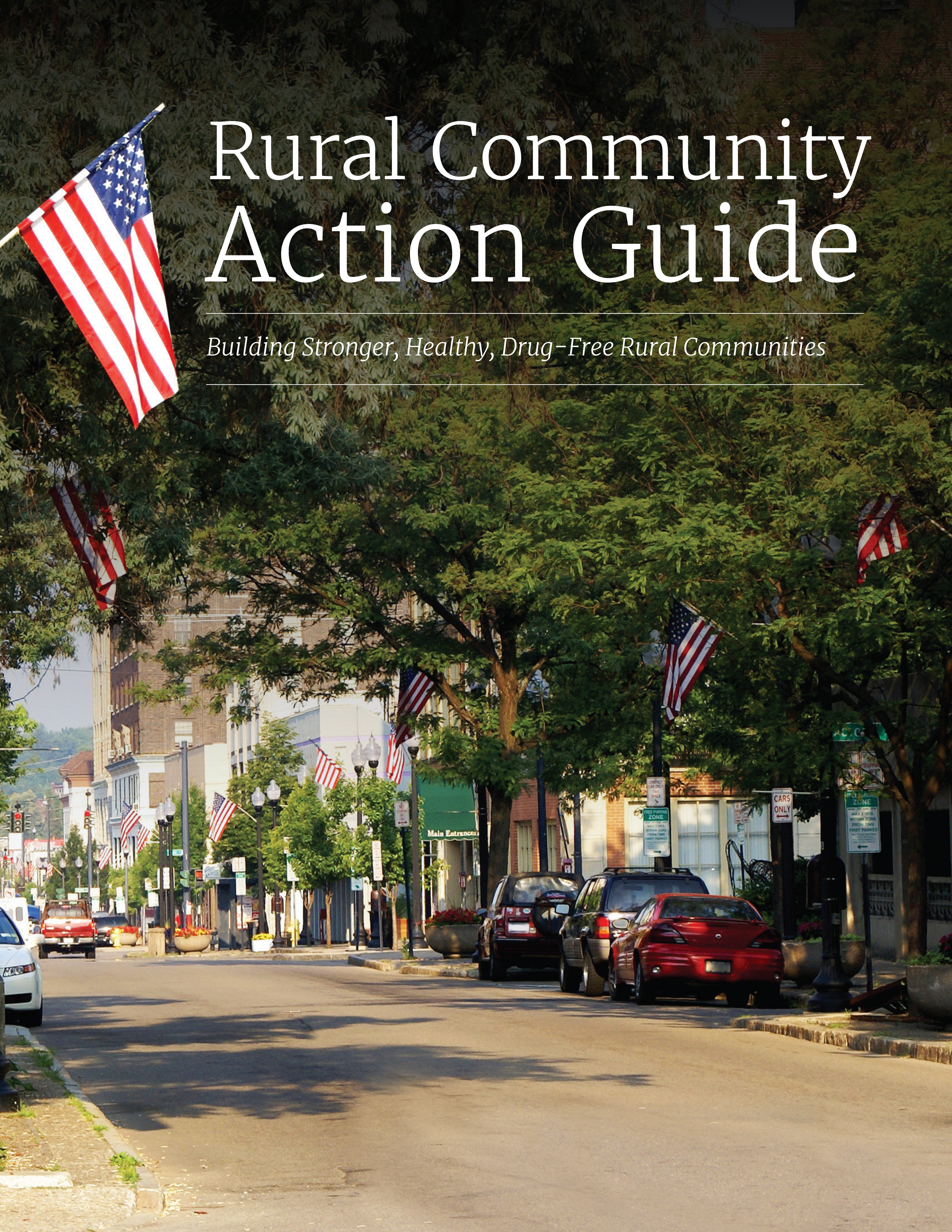

Inside Front Cover

Table of Contents

Letter from ONDCP Director, James W. Carroll 6

Letter from ONDCP Senior Advisor for Rural Affairs, Anne C. Hazlett 7

Abbreviations 8

Glossary of Terms 9

Introduction 11

SECTION 1: Face of Addiction 13

The Changing Face of Addiction 14

Moving Beyond Stigma in Rural Communities by Addiction Policy Forum 16

Using Data to Better Understand Substance Use Disorder and Opioid Use Disorder by NORC Walsh Center for Rural Health Analysis 20

SECTION 2: Impact of Addiction on a Rural Community 23

Managing the Push and Pull on Fiscal Resources during the Opioid Crisis by National Association of Counties 24

Workforce Development: Increasing Opportunities for Employment During the Opioid Crisis by National Association of Development Organizations 26

Rural Broadband is Fundamental to Increasing Healthcare Access in Rural Communities by The Rural Broadband Association 30

Bridging the Transportation Gap for Access to Substance Use Disorder Services in Rural Communities by National Rural Transit Assistance Program 34

Overcoming Economic Challenges Amid the Opioid Crisis by Appalachian Regional Commission 37

SECTION 3: Prevention 40

Stop Drug Use Before It Starts, Early Prevention and Early Intervention Strategies that Work by the U.S. Department of Agriculture, National Institute of Food and Agriculture 41

Community Driven and Shaped Solutions Change Lives by Community Anti-Drug Coalitions of America 46

Taking Action to Address Substance Use Disorder in the Farming Community
by American Farm Bureau Federation and National Farmers Union 49

SECTION 4: Treatment 52

Strengthening the Rural Healthcare Network for Persons Seeking Treatment
by National Rural Health Association 53

Understanding Why Medication Assisted Treatment is Different in Rural Communities
by The Pew Charitable Trust 57

Moving from Lock'em Up to Providing Treatment and Support
by National Sheriffs' Association 60

Making Drug Courts Work for Rural America by Center for Court Innovation. 63

SECTION 5: Recovery 69

How Faith Communities Can Help Rural Communities Address Substance Use Disorder
by the U.S. Department of Agriculture, Center for Faith-Based and Neighborhood Partnerships 70

Building a Strong Recovery Community in Rural Areas
by National Alliance for Recovery Residences. 73

Mobilizing the Recovery Community in Rural Areas by Faces & Voices of Recovery 77

Increasing Housing Options for Persons in Recovery in Rural Communities
by Housing Assistance Council 81

Closing Thoughts 84

Rural Community Action Guide Partners 85

Endnotes 90

Notice and Disclaimer:

The Rural Community Action Guide is a compilation of qualitative data collected from numerous community organizations, and data arranged by category. The guide aims to educate the public by providing an overview of the key challenges rural communities face when addressing the consequences of prescription opioid misuse and the use of illicit substances. It also showcases localized efforts implemented to help mitigate the impact of substance use disorder. The information provided herein should not be construed as the Federal Government's official policy position on these issues nor does it serve as an endorsement of the varying local practices included as examples throughout the guide. Further, this document is not intended to serve as legal advice, medical advice, or a substitute for seeking professional services. For more information on the Federal Government's policy position on Substance Use Disorder and Opioid Addiction, please consult the National Drug Control Strategy published by the Office of National Drug Control Policy.

Dear Rural Leader,

In small towns, suburban neighborhoods, tribal communities, and big cities, our Nation faces an unprecedented crisis of drug addiction. In 2017, more than 70,000 Americans, or nearly 200 people each day, died due to a drug overdose. And, while no corner of our country has escaped the devastation of this crisis, rural America has been particularly hard hit.

As the Nation's drug czar, I have seen firsthand the impact that addiction is having on small towns and rural places across our country. Traveling to places like Henryetta, a small farming and oil community in southeast Oklahoma, I have met with many local elected officials, law enforcement, healthcare providers, and families who have lost loved ones and seen the toll that drugs are taking on America's hometowns. In Henryetta and beyond, I have seen the drain of this crisis on already limited resources for public safety. And, I have visited rural places where there are no treatment options in the county, or even the surrounding region, to help people who are struggling with addiction.

To reverse course and ensure that rural America continues to be a place of prosperity for agriculture, manufacturing, and small business for generations to come, we must take bold action and do things differently than we have done in the past. We must be relentless.

Working with Federal, State, tribal, and local leaders, the Trump Administration has deployed an all-hands-on-deck approach to make critical resources available to rural communities. Thanks to these efforts, America saw the first decline in drug deaths in decades, with drug overdose deaths falling from their peak levels by as much as 24 percent in the hardest hit States like Ohio and Pennsylvania. Last year, my office, the White House Office of National Drug Control Policy, teamed up with the U.S. Department of Agriculture to compile a Federal rural resource guide to help rural leaders navigate the many Federal programs available to address the addiction crisis in small towns. In addition to funding, however, the Administration is helping rural communities with information to help create real change.

The Rural Community Action Guide is an important tool to equip rural leaders with critical information from lessons learned on the frontlines of prevention, treatment, and recovery in rural America. While no two rural communities are the same, there are promising practices gleaned from rural leaders in one town that can be replicated in another. With this information, local leaders can then design a more effective strategy for deployment in their own community.

As we step up to do even more to help small towns deal with the scourge of drug addiction, we are beginning to see signs of progress. Yet we know there is still much work to be done. In arming local leaders with tools like the Rural Community Action Guide, we will continue to be relentless in our attack and our commitment to serve as a strong partner to rural America in this fight. Together, we can defeat the crisis of addiction and build strong, healthy, and drug-free communities now and for generations to come.

James W. Carroll
Director of National Drug Control Policy

Dear Rural Partner,

From Montana to Maine, North Dakota to Texas, rural leaders are facing a monumental challenge: the crisis of drug addiction. Once thought to be a problem only in big cities, the addiction crisis is crippling small towns and rural places across rural America. No community, geography, or zip code has escaped its reach.

For rural communities, drug addiction poses a very real threat to the future prosperity of rural America. However, this challenge also creates an enormous opportunity for Federal, State, tribal and local leaders to set a new vision and build strong, healthy, and resilient places for the future. Working together as partners, we can save lives and keep our family, friends, and neighbors safe.

Over the past two years, I have had a chance to meet dozens of rural leaders who are on the frontlines of the response to this crisis. From the local judge who can't find enough foster homes for children at risk to farmers and rural business owners who are searching for a workforce, there are countless needs to be met. And, each community has different assets to be tapped in building a response.

Traveling to numerous small towns throughout rural America that have been impacted by drugs, I am frequently asked what I have seen that is making a difference. Local leaders are overwhelmed and looking for answers to help their communities in need. Beyond my own travels, I have also worked with many rural stakeholder leaders who, like me, are in constant search of lessons learned and activities from one community that can be replicated in another town.

The purpose of the Rural Community Action Guide is to arm rural leaders with information they can put into immediate action to create change. The urgency of this issue and its impact on quality of life and economic opportunity in rural America demands nothing less. Building strong, healthy, and drug-free communities will take all of us stepping up and playing our role at the Federal, State, or local level. With tools to build an effective response and a shared passion for the future of rural communities, we can defeat the crisis of drug addiction and create places of opportunity for today and hope for tomorrow.

Anne C. Hazlett
Senior Advisor for Rural Affairs
Office of National Drug Control Policy

Abbreviations

**BJA**	Bureau of Justice Assistance
**COAP**	Comprehensive Opioid Abuse Program
**DOJ**	Department of Justice
**DOT**	Department of Transportation
**ECHO**	Extension for Community Healthcare Outcomes
**EMS**	Emergency Medical Services
**EMT**	Emergency Medical Technician
**FDA**	Food and Drug Administration
**FQHC**	Federally Qualified Health Center
**HIV**	Human Immunodeficiency Virus
**HRSA**	Health Resources and Services Administration
**IHS**	Indian Health Service
**MAT**	Medication Assisted Treatment
**NAS**	Neonatal Abstinence Syndrome
**OTP**	Opioid Treatment Program
**OUD**	Opioid Use Disorder
**SAMHSA**	Substance Abuse and Mental Health Services Administration
**SUD**	Substance Use Disorder

Glossary of Terms[1]

**Addiction**	A chronic, relapsing disorder characterized by compulsive (or difficult to control) drug seeking and use despite harmful consequences, as well as long-lasting changes in the brain.
**Agonist**	A chemical substance that binds to and activates certain receptors on cells, causing a biological response. Oxycodone, morphine, heroin, fentanyl, methadone, and endorphins are all examples of opioid receptor agonists.
**Antagonist**	A chemical substance that binds to and blocks the activation of certain receptors on cells, preventing a biological response. Naloxone is an example of an opioid receptor antagonist.
**Buprenorphine**	An opioid partial agonist medication prescribed for the treatment of opioid addiction that relieves drug cravings without producing the high or dangerous side effects of other opioids.
**Drug Misuse**	The use of a substance for a purpose that is not consistent with legal or medical guidelines, most often with prescription medications. This could mean taking more than what is prescribed or taking a medication that was not prescribed.
**Medication Assisted Treatment**	Treatment for opioid use disorder combining the use of medications (methadone, buprenorphine, or naltrexone) with counseling and behavioral therapies.
**Methadone**	A long-acting opioid agonist medication used for the treatment of opioid addiction and pain. Methadone used for opioid addiction can only be dispensed by an OTP certified by SAMHSA and approved by the designated State authority.
**Naltrexone**	A long-acting opioid antagonist medication that prevents receptors from being activated by other opioids. Naltrexone is used to treat alcohol and opioid use disorders.

**Naloxone**	An opioid antagonist medication approved by the FDA to reverse an opioid overdose. It displaces opioid drugs, such as morphine or heroin, from their receptor and prevents further opioid receptor activation.
**Neonatal Abstinence Syndrome**	A condition of withdrawal that occurs when certain drugs pass from the mother through the placenta into the fetus' bloodstream during pregnancy causing the baby to become drug dependent and experience withdrawal after birth.
**Opioid**	Natural or synthetic chemicals that interact with opioid receptors on nerve cells in the body and brain and reduce the intensity of pain signals and feelings of pain. This class of drugs includes the illegal drug heroin, synthetic opioids, such as fentanyl, and pain medications available legally by prescription, such as oxycodone, hydrocodone, codeine, morphine, and many others.
**Opioid Use Disorder**	A problematic pattern of opioid use that causes significant impairment or distress.
**Prescription Opioids**	Medications used to treat moderate to severe pain often prescribed following surgery or injury.
**Prescription Drug Misuse**	The use of a medication in ways or amounts other than intended by a doctor, by someone other than for whom the medication is prescribed, or for the experience or feeling the medication causes.
**Substance Misuse**	The use of any substance in a manner, situation, amount, or frequency that can cause harm to users or to those around them.
**Substance Use Disorder**	A medical illness caused by disordered use of a substance or substances. According to the Fifth Edition of the Diagnostic and Statistical Manual of Mental Disorders (DSM-5), SUDs are characterized by clinically significant impairments in health, social function, and impaired control over substance use and are diagnosed through assessing cognitive, behavioral, and psychological symptoms. An SUD can range from mild to severe.

Introduction

The core function of the Office of National Drug Control Policy (ONDCP) is to develop Federal drug policy and coordinate its implementation across the Federal Government. The 2019 National Drug Control Strategy (herein referred to as Strategy) sets out the President's priorities for reducing drug use and its consequences, now and in the coming years. Importantly, in addition to reducing illicit drug availability through law enforcement and interdiction efforts, the Strategy provides strategic direction for the Federal Government's efforts to prevent drug use before it starts, identify substance use disorder and intervene early, expand access to evidence-based treatment, and provide the ongoing recovery support Americans need to achieve and sustain recovery.

The Rural Community Action Guide complements the Strategy by providing topical insights on how rural leaders can and are addressing drug use and its consequences to build strong and healthy rural places. Developed with input from a series of roundtables on opioid misuse hosted by the U.S. Department of Agriculture, and key partners, this comprehensive report presents strategies to strengthen the local responses to addiction in rural communities and fresh ideas to adopt flexible, creative solutions at the local level.

"The *Rural Community Action Guide* provides topical insights on how rural leaders can and are addressing drug use and its consequences to build strong and healthy rural places."

Rural communities face many hurdles in addressing opioid and other drug addiction. Research shows that people living in rural America who need help are falling through the cracks, often losing their lives. Treatment services are insufficient to meet rural demand. Access to quality medical care, resources, and training is limited in rural communities, particularly for specialized populations, such as pregnant women, parents, and seniors. Further, drug courts, which are known to be significantly more effective than incarceration for non-violent offenders, are not available in many rural areas.

Between 1999 and 2015, drug overdose deaths in rural counties jumped by 325 percent[2] as compared to 198 percent in metropolitan areas. Last fall, Harvard University[3] released a survey of rural Americans who identified drug addiction as the biggest challenge facing their community. And, in 2017, two leading farm groups[4], the American Farm Bureau Federation and National Farmers Union, conducted a survey that found nearly 50 percent of rural Americans, and 74 percent of farmers, have been directly impacted by opioid misuse. In the face of these challenges, rural leaders are working hard in collaboration with government and private sector partners to improve SUD outcomes while building strong and healthy rural communities

Stigma prevents many people in rural America from seeking and accepting help. Once people get treatment, there is a substantial need for better connection to recovery support, such as stable housing, employment and job training, education, medical services, transportation, and childcare in a manner that takes into account the infrastructure challenges inherent in many rural places. In addition, local leaders need greater access to timely and accurate data about substance use disorder in their community, including information about upstream drivers that are causing higher rates of substance use.

The Administration has deployed an all-hands-on-deck approach to make critical resources available to our local communities. In May 2018, ONDCP stood up the Rural Opioid Federal Interagency Working Group to help address the opioid crisis by improving coordination and reducing potential overlap among Federal agencies responding to the crisis in the Nation's rural communities. The group compiled a **Federal rural resources guide** to equip rural leaders with a comprehensive directory of the many Federal programs available to help small towns address the opioid crisis.

This guide continues the effort to support rural leaders by providing insight and recommended action steps from partners who are working on the frontlines of combating drug addiction in rural communities. The direction offered in this guide is then supplemented by examples of promising practices which can be found on the **USDA webpage**. With this information, local leaders will be better equipped to take action on the ground. ONDCP extends its sincere gratitude to the many individuals, organizations, and stakeholders who shared personal stories and offered thoughtful input to the development of this resource.

SECTION 1

Face of Addiction

PARTNER INSIGHT AND RECOMMENDED ACTION STEPS

To improve SUD outcomes and build strong and healthy communities, rural leaders must first change how they understand, talk about, and respond to addiction. This fundamental shift often begins with understanding the face of addiction, meaning who is impacted by SUD, in the community. The scope of its impact can best be understood by personal stories and data.

Once that collective understanding is built, community leaders are better able to recognize the signs of addiction and break down stigma. Stigma is one of the major roadblocks that keep people from seeking and accepting treatment for SUD. The impact of stigma is often magnified in a rural community where relationships are close-knit and there is less privacy.

In addition to breaking down stigma, increasing access to data is critical to answering many questions about addiction and rural communities. Data can help local leaders better understand who is being impacted, what the greatest needs are at both an individual and community level, and what will effectively keep people healthy.

THE CHANGING FACE OF ADDICTION

Today, nearly every community has been affected by drug addiction. As the prevalence of SUD and drug overdose deaths continues to rise in rural areas, drug use can no longer be viewed as an urban and inner city problem. Beyond zip code, drug addiction is impacting individuals and families from all socioeconomic groups, changing the image of a person with SUD. Someone struggling with addiction could be a working mom, senior citizen, pastor, coach, high school athlete, or college student.

In 2018, USDA convened a series of rural conversations about opioid misuse to raise awareness and to better understand the impact of this crisis on small towns. At each event, a local person in recovery shared his or her journey with addiction and road to sobriety. Below are five stories that illustrate the ever-widening scope of people in rural America who have been affected by addiction.

"Someone struggling with addiction could be a working mom, senior citizen, pastor, coach, high school athlete, or college student."

Alexis (Pennsylvania)

The youngest of nine children, **Alexis Johnson** never drank, smoked, or used drugs all through high school, earning exemplary grades and entrance to Penn State University. While attending Penn State, she tried ecstasy and cocaine because she just wanted to fit in and be a part of something after joining a sorority. Alexis graduated in 2005 with an A average in criminal justice, a double minor in sociology, and with every intention of going to law school. Three DUI charges later, she had moved from pain medication to heroin and had overdosed seven times. Now in recovery, Alexis wants to see "better after care programs" for persons with SUD who complete treatment, observing that people "complete these programs and then go back out into the streets with no life skills." She is now a motivational speaker and aspires to work with teens in prevention.

Dale (Utah)

Since boyhood, **Dale Covington** wanted to work at Hill Air Force Base near Salt Lake City, Utah. He described himself as a lonely man, a depressed man, a depressed father, and a depressed husband who needed people to come to him and say, "there is still hope within you." Twelve years ago, his wife and four kids aged four to 16, met him at the door and said, "Dad, we love you, but unless you seek help, we will help you pack." Now in recovery, he acknowledges an ongoing journey. "I still struggle with the temptation each and every day. I would not be a part of their lives if I would not have adapted to programs that helped me, that gave me hope." Their support, coupled with coworkers who treated him with compassion, reached him at his lowest point. Dale joined a treatment program and moved into long-term recovery, later helping steer hundreds of persons in recovery toward resources along their

journey to sobriety.

Alex (Kentucky)

Alex Elswick grew up in a healthy, happy Kentucky home without neglect, abuse, divorce, or alcoholism. As a youth, he struggled with anxiety. At age 15, he experimented with marijuana and his anxiety dissipated, helping him feel normal. His addiction surfaced when he was prescribed Oxycontin after wisdom teeth surgery at age 18. In the fall of 2012, he was in college studying for law school, and nine months later, after four treatment centers, he spent his last weeks of addiction homeless, living under a bridge in Dayton, Ohio, begging for money, and injecting heroin. After getting treatment, he had to confront the aftermath of his addiction including a criminal record, bad debt, and a poor credit rating. Now in recovery, Alex intentionally works to “recover out loud” and to never lose sight of the suffering people and hurting families impacted by addiction. Through a non-profit group, Voices of Hope, Alex and his mother provide telephone support to people in recovery and advocate for needs in the recovery community.

Kent (Oklahoma)

University of Oklahoma chaplain **Kent Bowles** served in ministry for more than two decades. He was a model citizen with a wife and children. Yet opioids took over his life, initially prompted by medical conditions. It was several years into his addiction, before he classified or categorized himself as a person with OUD. Because of the role he served and the life he lived, he thought he could not be that person struggling with addiction. One pill became two, became three, became a weekend, became a day, became night and then morning. He was in a position where the access was very easy because he was a person who was trusted and did not look the part of someone who was addicted. Kent clearly recalls the day he was arrested as “the most painful day of my life,” but it also became the most powerful opportunity for recovery. He founded the What’s Important Now (WIN) Foundation and serves as a connecter, networker, and recovery guide providing conversation, personal visits, transportation, and referrals.

Courtney (Maine)

The week before her twenty-second birthday, **Courtney Allen**, overdosed three times. She began using substances at age 13 and by age 19, Courtney was using drugs intravenously. For a decade, she struggled with poverty, trauma, and addictions to alcohol, heroin, Ritalin, and cocaine. On February 8, 2015, her life changed. Afraid that her young children would end up like her, she checked into an Augusta detox center for help and agreed to participate in the Augusta Family Treatment Drug Court, a civil court that assists parents working toward reuniting with their children. In her words, she found help from one of the least likely places she thought would work—the court. She received intensive counseling and wraparound services. After a year of treatment, she regained custody of her children and enrolled in school. Next spring, she will graduate at the top of her class with a BA in Social Justice and looks forward to continuing her education in graduate school. Her free time is spent advocating for resources to fight SUD and the opioid crisis.

MOVING BEYOND STIGMA IN RURAL COMMUNITIES

By Addiction Policy Forum

STIGMA ACTS AS A BARRIER TO OBTAINING SUBSTANCE USE DISORDER SERVICES

Fear of stigma is one of the major roadblocks to accepting services for SUD, leading to poor health outcomes for patients. Stigma, the sense that something is shameful, may be felt more acutely in small rural towns because of the relative lack of anonymity. In a city, neighbors are less likely to know if a person is seeking substance use services or going to get some help. In a small town, it is more likely that people will know that a person visited a certain provider for help.

Stigma can inhibit a person's access to treatment and positive recovery outcomes, alienate their families who fear judgment and isolation, and create unnecessary division in tight-knit rural communities. The difficulty of maintaining anonymity while engaging with treatment can cause a person with SUD to stop or avoid seeking treatment altogether. Due to the stigma surrounding SUD and a general lack of understanding about the disease of addiction, patients who are treated for SUD or in recovery may face discrimination in the courts, the workplace, and healthcare facilities, especially in rural communities with scarce treatment and recovery resources.

According to a study conducted by SAMHSA in 2014, more than 20 percent of people with SUD did not seek treatment because they worried about the negative impact on their employment, and more than 17 percent were concerned about how their community would view them.[5] The extent of misinformation about SUDs in public discussion—especially among healthcare providers, educators, policymakers, and media—reinforces barriers that prevent people from seeking help.

SCIENCE OF ADDICTION

Although 20 million Americans struggle with SUD,[6] about half of the population does not think that addiction is a disease[7]. Misunderstandings about addiction are often propagated by a limited understanding about neurological science. This is one reason addiction is often not handled like other chronic illnesses, medical research is impeded, and counterproductive myths about treatment are perpetuated.

> "Stigma will fade in the face of education about the negative impacts of untreated addiction on families and communities."

Addiction is a chronic, relapsing disorder characterized by compulsive substance seeking and use despite harmful consequences.[8] Brain scans show that SUDs affect tissue function in two main parts of the brain: the limbic system and the cortex. The limbic system rewards completion of basic survival tasks, such as eating and finding shelter, by releasing dopamine. Substance use activates this dopamine release much more powerfully, and repeat occurrences can condition the brain to believe the substance is necessary for survival. SUDs also disrupt the cortex, which houses impulse control and decision-making abilities, by eroding the self-control and discipline required to make prudent choices, while

simultaneously producing intense impulses to use substances again.

The good news is that SUDs are both preventable and treatable. Brain scans show that parts of the brain impaired by addiction can improve during recovery. Given access to proper treatment and support, individuals with a SUD can transform their lives, influence their environments, and realize positive health outcomes.

Rural communities must also change how they understand, discuss, and respond to addiction if they are to win the fight against America's spreading opioid crisis. Stigma will fade in the face of education about the negative impacts of untreated addiction on families and communities and increase the likelihood that those who need help can access it.

RECOGNIZE THE SIGNS OF ADDICTION

Prevention begins with the observation of common danger signs. Know what to watch for and how to respond compassionately to those struggling with the isolating, confusing, and painful experience of SUD.

Red Flags

- Disinterest in activities that were once enjoyed
- Changes in mood and/or daily routine
- Decline in performance at work or school
- Secretive behaviors
- Change in weight or appearance
- Inability to be present in conversation

Tips

- Open and empathetic communication without blame or accusation works most effectively.
- Always make sure the person is not a harm to others or self.
- Early connection to resources and education can make all the difference.

MODIFY LANGUAGE TO HELP ADDRESS STIGMA

Being thoughtful about the language we use to talk about addiction is an important step in reducing the stigma surrounding SUD. Words like "addict" and "junkie" negatively and inaccurately portray addiction as a choice and identity. Referring to a "person with SUD" more accurately describes addiction as a medical condition that can be treated.

Harmful Terms	Positive Terms
Junkie/Addict	Person with a substance use disorder, (opioid use disorder, alcohol use disorder)
Drug habit/drug misuse	Substance use disorder
“clean” (a “negative” toxicology screen)	Not currently using substances
“dirty” (a “positive” toxicology screen)	Currently using substances
Replacement/Substitution Therapy	Medication Assisted Treatment refers to the use of any medication approved to treat substance use disorders combined with psychosocial support services

ACTION STEPS TO COMBAT STIGMA IN RURAL COMMUNITIES

Stigma is one of the primary reasons that many do not take that critical step toward addressing their SUD—asking for help. Community leaders can help to address this challenge by engaging in the following four activities:

1. **Understand the science**. Decades of research have given us the tools to improve our response to addiction and empower communities to help prevent, treat, and support those in recovery from SUDs. Use science to debunk stigma and support local policies and practices that promote a science-informed response to addiction. Visit the National Institute on Drug Abuse at **drugabuse.gov** or SAMHSA at **samhsa.gov** to learn more.

2. **Educate the community about SUDs and opioid misuse**. Addiction does not discriminate and can impact anyone (family members, friends, and peers). Use statistics and the latest science to teach the community about addiction and work with stakeholders in the community to disseminate knowledge and resources like overdose reversal kits, the brain science of addiction, and other educational materials. Find free resources by visiting **addictionresourcecenter.org**.

3. **Develop and implement a marketing plan to reduce stigma**. The best way to counter inaccurate stereotypes or myths is to replace them with information. Anti-stigma marketing can present facts on SUDs with the goal of correcting misinformation or contradicting negative attitudes and beliefs. The communication strategy should also be tailored to the audience. Social media and online platforms may be more appropriate communication streams for young people, while a talk at the local Rotary club or having a public information ad campaign and printed pamphlets may be more appropriate for adults.

4. **Share positive stories of recovery**. When people in recovery from SUD share their stories, the stigma is diminished. Community leaders should facilitate and support efforts to build the capacity of the recovery community to participate in public dialogue about addiction, treatment, and recovery.

USING DATA TO BETTER UNDERSTAND SUBSTANCE USE DISORDER AND OPIOID USE DISORDER

By NORC Walsh Center For Rural Health Analysis

More than 70,000 people in the United States died from a drug overdose in 2017, and approximately two-thirds of those deaths involved opioids, based on CDC data[9]. Addressing SUD is a national challenge, but the solution lies at the local level. Rural America needs to be part of the overdose discussion because the stakes are so high there, yet rural research presents unique challenges. Data collection in rural areas is difficult due to both small numbers and the frequent reliance on survey data with insufficient sample sizes to allow for rural specific analysis.

Data can help rural leaders respond to the big questions—what causes the use of illicit substances in their communities, what perpetuates the problem, and what will mitigate the problem. After identifying the problem and its impact on the larger community, rural leadership can also utilize data to uncover solutions, including prevention, treatment, and recovery options for each community. Using data will help communities and leaders in the following three important ways.

"Rural leadership can also utilize data to uncover solutions, including prevention, treatment, and recovery options for each community."

Understand the changing face of the crisis

Both prevalence of OUD and deaths from drug overdoses continue to rise among people of all genders, races, and ages. The opioid crisis impacts individuals living in both urban and rural areas across the country. However, rates of drug overdose deaths have been increasing in rural areas, and in 2015, the rural rate surpassed the urban rate[10]. The crisis has continued to expand and impact growing numbers of people from diverse communities. In many rural communities, a large portion of population has been directly affected in some way. Strong data systems are needed to continue to track communities most affected by OUD and drug overdose deaths.

Better understand the impact on the local community

Increased access to data that identifies the problem at a local level is critical to help local leaders understand the impact on rural communities. The opioid crisis has strained healthcare providers, local economies, law enforcement, emergency response, and social services, including the foster care system and schools. It has also amplified the need for broadband and rural transportation systems. Recruiting and retaining businesses can be difficult in regions with significant burden of the opioid crisis, which

can create cascading effects in terms of overall poverty in these communities.

Identify prevention, treatment and recovery models that work

Data on individual recovery success reveals the effectiveness of a community's overall response to the opioid crisis. Initially, data and evidence-based research reveal the types of prevention, treatment, and recovery models that are most effective for different populations within a particular community. Continuing to collect data on interventions also helps build the evidence-base for successful prevention of opioid misuse. Data is critical to identify where access to treatment options is limited, often the case in many rural communities. Understanding where current treatment resources are available also helps local communities decide where additional resources need to be allocated. When leadership understands where the burden of opioid misuse and mortality is highest, they can more prudently invest adequate resources in local infrastructure, treatment, and emergency services.

> "The Community Assessment Tool demonstrates the utility of data visualization in support of community health planning."

THE COMMUNITY ASSESSMENT TOOL

The Community Assessment Tool (**CAT**)[11] developed by NORC in partnership with USDA provides local stakeholders, policy makers, and residents with access to overdose mortality data for their communities. CAT users can also learn about factors that provide additional context around opioid addiction and death, including the strength and diversity of local economies, race, ethnicity, educational attainment, and disability status of residents. This tool puts overdose statistics in socioeconomic context, delivering added value to communities seeking to develop comprehensive strategies that address the crisis.

CAT demonstrates the utility of data visualization in support of community health planning and response. Insights can be used to target resources and interventions for communities that most need support. By allowing those on the front line to access local data and compare rates to those across their States and across the nation, CAT users can better understand the magnitude of the opioid crisis in their communities, as well as trends over time. By overlaying socioeconomic and demographic information, CAT users can also begin to consider local factors that may contribute to higher rates in their communities. Armed with this information, stakeholders and policy makers can begin a dialogue to increase understanding and to begin the process of identifying and implementing local solutions.

Overdose mortality rates are provided for two time periods, 2008-2012 and 2013-2017, which allows CAT users to see a visual representation of how the data has changed over time within the borders of each county on a national map. Additionally, CAT users can zoom into specific States or regions and move the cursor over a county to show overdose mortality rate information or create a data fact sheet about the county, overdose mortality data, socioeconomic and demographic data, and how the county compares with the State and the nation. The tool delivers access to user-friendly and relevant data that assists rural leadership in building grassroots solutions for prevention, treatment, and recovery.

ACTION STEPS TO USE COMMUNITY DATA BETTER

Communities looking to improve the way data is used should:

1. **Determine the magnitude of the problem in the community**. Many communities still don't understand the extent of the SUD problem in their region. Communities should use the CAT as a starting point to learn how their local rate of drug-overdose deaths compares to state and national data. It provides access to user-friendly and relevant data to assist them in building grassroots solutions for prevention, treatment, and recovery.

2. **Begin a community dialogue to inform and engage leaders and build grassroots solutions**. The CAT allows users to see a visual connection between overdose numbers and socioeconomic factors. It does not provide specific answers or solutions; it provides information that can be used to drive the conversation and spur tailored action.

3. **Consider whether more resources are needed**. Determine when current resources are sufficient to address the problem or whether additional resources are needed. Implement interventions and ensure mechanisms are in place for ongoing data collection and evaluation of effectiveness.

4. **Build community coalitions to help drive solutions**. Rural leaders should support and develop technical assistance resources to assist in implementing broad-based coalitions. Learn from other regions, evaluate the effectiveness of community coalitions, and use the results to develop a model and targeted strategies specific to the issues in their rural community.

SECTION 2

Impact of Addiction on A Rural Community

PARTNER INSIGHT AND RECOMMENDED ACTION STEPS

Drug addiction in rural America is more than just a health issue. This is a matter of rural prosperity. A 2017 report by the White House Council of Economic Advisors estimated that the impact of the opioid crisis on the U.S. economy in just one year was more than $500 billion dollars. This crisis is impacting worker productivity, increasing healthcare demands, and putting enormous stress on already limited resources for emergency response, law enforcement, and social services. This crisis is also making economic development even more difficult for rural communities, which often struggle to attract new investment.

With that impact, rural leaders must develop the knowledge, capacity, and infrastructure to effectively deliver the resources needed to build a strong and healthy community. An effective local response will encompass resources to meet a wide range of needs at both an individual and community level, such as healthcare, public safety, workforce training, rural broadband, transportation, and economic development. Rural leaders are best positioned to meet these needs and maximize the impact of taxpayer dollars invested when they coordinate and collaborate with neighboring jurisdictions as well as Federal, State, and non-profit partners.

MANAGING THE PUSH AND PULL ON FISCAL RESOURCES DURING THE OPIOID CRISIS

By National Association of Counties

CHALLENGING FISCAL FACTORS IN RURAL COUNTIES

Rural counties in America are making progress on the ground level in the fight against opioid addiction, but the costs have been high and are mounting. Counties work on the frontlines to advocate for and protect the health and safety of residents affected by the opioid crisis. Counties directly experience the human toll and shoulder a large share of the costs required to respond to the crisis. They deliver key prevention, treatment, and recovery resources for SUDs through local agencies, as well as public safety resources and emergency first response teams. Due to the increased demand for services resulting from the opioid crisis, local governments are acutely feeling the strain on resources, from a shortage of hospital beds to overworked foster care systems and overcrowded jails.

As a result, local governments are struggling to contain the scope and costs of the opioid crisis in their jurisdictions and provide adequate addiction response and recovery support services. Not surprisingly, handling the different challenges that flow from the opioid crisis is straining county resources. The national opioid crisis has been playing out most markedly in remote, rural areas, which presents a challenge for healthcare providers already hindered by rising healthcare costs. In rural counties the costs can be attributed to services surrounding overdose deaths and burdens to the criminal justice system.

REDUCING COSTS IN RURAL COMMUNITIES

Leaders in county and city governments are exploring innovative strategies to address the fiscal strain of dealing with the ongoing opioid crisis. In 2016, Boulder County established an Opioid Advisory Group in Colorado to improve the community-based response to the crisis through coordination across county medical providers and law enforcement officials, and in tandem with harm-reduction and faith-based organizations in the county. In the two years since the group's creation, the county reported a 36 percent decrease in opioid prescribing at the county's largest hospital and 191 lives saved by naloxone administered by community members and law enforcement.

"Counties directly experience the human toll and shoulder a large share of the costs required to respond to the crisis."

Other locally-led programs are meeting patient needs on an individual level. In New Jersey, Ocean County launched the "Recovery Coach Program," a voluntary initiative connecting individuals who are revived by naloxone with treatment options once they are stabilized in emergency rooms. In cooperation with local hospitals, the program matches overdose victims with recovery coaches. If the patients are willing, coaches provide mentoring for up to eight weeks and help guide them toward recovery. Treatment is free or partially subsidized for those willing to participate. Program coaches are often working through the recovery phase of addiction themselves and can add a valuable perspective to the individual's recovery.

Local leaders are finding resourceful and impactful ways to confront the opioid crisis in their jurisdictions thereby helping to relieve the strain on local services. However, as the Nation seeks to bring an end to the crisis, cities and counties recognize that these efforts are more effective when carried out in,

partnership with State, Tribal and Federal counterparts. The National Association of Counties has been working diligently toward this goal in recent years with intergovernmental partners to emphasize the importance of expanded prevention, treatment, and recovery resources. They are also identifying policy recommendations for Federal, State, and local officials that could maximize the intergovernmental response to opioid overdoses and deaths.

ACTION STEPS TO MANAGE FISCAL RESOURCES DURING THE OPIOID CRISIS

As rural community leaders look to build healthy and prosperous communities and deliver services while dealing with the opioid crisis, they must maximize government efficiency and ensure responsible stewardship of taxpayer dollars. Here are four ways to manage the crisis more efficiently at the local level:

1. **Cut off illicit traffic**. Stop the problem at the source. Work with Federal, State, and local partners to reduce the supply of illicit opioid analogues, including fentanyl and carfentanil. These synthetic forms of opioids are becoming a leading cause of overdoses in communities as drug traffickers lace drugs with stronger opioids to create a more potent product.

2. **Calculate the total cost**. Start adding up the costs associated with the opioid crisis in your region. The crisis consumes enormous fiscal resources in small counties and building awareness of that drain will motivate support and generate action within the community. Quantifying the causes and consequences of the crisis will help leaders develop and implement effective prevention, mitigation, and intervention policies.

3. **Consider new alternatives**. Look for new ways to approach needs. Counties can improve health and addiction treatment services for justice-involved individuals and embrace alternatives to punishment models to decrease short- and long-term healthcare, disability, and criminal justice costs to both local taxpayers and the Federal Government. Invest in technology to help communities work better and smarter and more efficiently.

4. **Cross-collaborate to cut costs and improve efficiency**. Solutions are more effective when coordinated among the various governments within a region. Explore ways to cross-collaborate to help minimize costs. Initiate regional cooperation and strengthen lines of communication with neighboring governments. By securing partnerships with the public, private, and nonprofit sectors, counties remove barriers, overlap and duplication, and better coordinate service delivery. Pursue public and private funding opportunities to bring programs and initiatives to the community. Engage with all critical community stakeholders and connect local efforts and community working groups with State and county efforts to make sure that the community is implementing coordinated responses.

WORKFORCE DEVELOPMENT: INCREASING OPPORTUNITIES FOR EMPLOYMENT DURING THE OPIOID CRISIS

By National Association of Development Organizations

RURAL BUSINESSES FACE UNIQUE CHALLENGES

Rural businesses typically face unique challenges to their success, such as limited educational facilities, scarce professional training programs, and less than optimal infrastructure. The widespread use of illicit substances and the consequences of prescription opioid misuse in rural communities has ushered in new and unexpected problems for rural employers. It often leads to cycles of unemployment for many prime-age workers in rural communities and long-term, severe consequences for both workers and businesses.

A rural community's economic and social well-being is also tied to worker engagement. A strong, healthy workforce promotes business and encourages local development. High employment propels local economic activity, which in turn expands city and county revenue for public services and programs. Additionally, high workforce engagement supports community-based activities through faith-based, civic, and nonprofit organizations. Alignment of people and resources to address needs provides greater leverage leading to better outcomes for communities overall.

The most profound challenge for businesses associated with SUD is the difficulty in filling open positions with workers who can pass a standard drug test. Other challenges lie primarily in attracting and maintaining an adequate and consistently productive workforce. Finding ways to support employees who develop a substance use problem while on the job and retain the employees who struggle to maintain sobriety. Finally, finding ways to prevent employees without a SUD from falling into addiction and expanding workplace practices to include people in recovery from substance misuse.

"The most profound challenge for businesses associated with SUD is the difficulty in filling open positions with workers who can pass a standard drug test."

WORKPLACE SAFETY AND ADVERSE CAREER EFFECTS

Businesses experience poor productivity from employees coping with SUD because the ability to provide dependable product and services rests on the reliability of staff and consistent operations. Business cannot act effectively without consistent employee support, and employees grappling with substance misuse usually underperform or fail to perform adequately in the workforce. Likewise, employee recruitment rates suffer in a community hit by the opioid crisis. Initial drug tests disqualify

several prospective hires during the screening process. Available personnel undergoing recovery also present uncertainty because of their ability to meet job responsibilities.

Workplace safety is the highest priority for employers. Workers under the influence can jeopardize that commitment, presenting danger to themselves and others. Employers must maintain adequate staff to operate a business and may not have the luxury of giving struggling personnel time to fully recover from SUD. Workers with SUD often struggle with commitment to their employer and ability to perform tasks, especially at the incremental levels necessary to advance in their careers. Workers who underperform or prove inconsistent in job functions cannot be trusted to carry out roles and responsibilities of their positions. These disadvantages put rural businesses at risk against urban and global competition that may employ stronger teams with better resources and capable of greater innovation and productivity.

THE ROLE OF EMPLOYMENT IN RECOVERY

Counties across the country are realizing that a quality workforce is one of the single most important factors for promoting county economic competitiveness. To be successful after treatment, people in recovery need to be part of the community—and this usually means being employed. Employment can aid those in recovery by providing purpose in their lives, establishing a reassuring sense of routine, and integrating structure that replaces negative decisions with positive choices.

However, for a person in recovery, returning to work comes with various barriers that extend beyond the State of the job market, the level of education, or qualifications, such as stigma in the community, impaired health, and an ever-present struggle to maintain sobriety. Finding a job after rehabilitation or during recovery may also mean making some sacrifices to prioritize that person's sobriety, such as working part-time or alternative hours in order to accommodate treatment and meetings. If that person has been displaced from the workforce for some time, reentry into the job market may mean taking a lower paying opportunity.

The challenge is finding more quality programs in rural areas that connect people in recovery to employers willing to give this pool of potential employees a second chance. Employers may demonstrate empathy and support for people in recovery yet doubt the candidate's ability to effectively and consistently carry out work responsibilities. The person in recovery needs an opportunity to show that he or she can be accountable and productive in the workplace and maintain his or her sobriety.

"The person in recovery needs an opportunity to show that he or she can be accountable and productive in the workplace and maintain his or her sobriety."

RURAL ECONOMIC COLLABORATION WORKS

When rural leaders and business owners work together with healthcare providers and law enforcement, the community can collaboratively develop innovative solutions for connecting people in recovery to economic resources and opportunities. Collective engagement acknowledges the severity of this epic public health issue and utilizes resources more effectively than separately.

Employee recruitment and workforce training are two of the most difficult business development challenges for small communities. Ostracizing those in recovery only limits the pool of available workers. A community-wide support system led by employers and public service organizations can offer support to job seekers in recovery. Additionally, local and regional educational opportunities and skills training benefit both employers and employees. Workforce boards and related training programs can adopt curriculum and methods that acknowledge the effects of addiction. Service providers can better assist those in recovery to gain skills and improve job prospects.

> "A community-wide support system led by employers and public service organizations can offer support to job seekers in recovery."

For example, Pennsylvania community leaders joined together to form Somerset County Recovery and Reentry Center, which connects treatment, skills training, and employers to support job seekers in recovery. The initiative grew out of a lack of qualified workers and the community's desire to help people with a SUD with sustainable employment. The program relies on a strong partnership between area employers, treatment providers, law enforcement, and the workforce development board. Participants in the program follow guidance provided by addiction counselors in various stages from skills training to employment. Employers accept people enrolled in treatment and training who maintain good standing. Many employers even provide compensation through training and skills development for eligible workers in their respective fields.

Engagement on SUDs requires comprehensive awareness of the health, environment, economic, and social aspects of this complex issue as well as the community impact. For example, in West Virginia, Mercer County's assessment of its economic and social status revealed a number of issues specific to the locality, impacting economic development, community well-being, and overall quality of life including the use of illicit substances and prescription opioid misuse.[12] Broad-based assessments of this type allows employers to better understand the root causes of addiction and can help increase local business efforts to prepare outreach and skills training for job seekers in recovery.

ACTION STEPS TO INCREASE EMPLOYMENT OPPORTUNITIES

Local leaders and employers should consider the following steps to increase employment opportunities for people with SUD:

1. **Invest in training to help employers and staff recognize the signs of addiction**. Addiction is a disease with a range of harmful conditions and behaviors. The signs and symptoms of SUD can vary with the individual. Consider training for managers and supervisors so they can identify the early warning signs. Recognizing these signs can help a person with SUD receive the treatment they need.

2. **Encourage recovery friendly workplace initiatives**. Give the business community and employment organizations the resources and support they need to foster a supportive environment for people in recovery. Encourage initiatives that support community health and wellness and encourage those in addiction recovery to devote themselves to positive activities. Community recreation centers and parks, exercise classes and fitness clubs, diet and nutrition programs, and other wellness services can all promote healthy lifestyles and deter people from substance use.

3. **Support second chance initiatives**. Reentering the workforce is a key barrier preventing people in recovery from transitioning to a more productive lifestyle. A person in recovery has the potential to be a great employee. Test innovative approaches and support employers that develop second-chance policies and hire individuals in recovery.

4. **Engage the employment sector early**. Every aspect of the community is impacted by substance use. Businesses are particularly critical partners to support workforce development. Rural employers need to understand the path from addiction to recovery and the role employment plays in recovery. Better understanding of the dynamics of addiction recovery could dispel myths about people in recovery and inspire support for second chance initiatives.

5. **Build partnerships and coordinate to leverage resources**. Collaborative partnerships in the public and private sector are necessary for successful implementation of plans to combat opioid crises in rural communities. Different programs and services help a person in recovery become "work ready" (e.g., recovery-oriented services, peers support and mentoring, career development, etc.). Employers can build trust and minimize risk by partnering with different stakeholders and businesses.

RURAL BROADBAND IS FUNDAMENTAL TO INCREASING HEALTHCARE ACCESS IN RURAL COMMUNITIES

By The Rural Broadband Association

CONNECTIVITY SPURS RURAL PROSPERITY

Reliable and affordable broadband is a catalyst for rural prosperity that unlocks economic development, innovation, advancements in technology, workforce readiness, and an improved quality of life. Modernizing infrastructure, building partnerships, and driving innovation is the key to success in strengthening e-connectivity across the rural heartland. Most importantly, e-connectivity in rural areas can save lives when public health disasters strike, especially complex occurrences like the opioid crisis.

> "Reliable and affordable broadband is a catalyst for rural prosperity."

When the President declared the opioid crisis a nationwide public health emergency in October 2017, many agencies began gathering critical resources and implementing innovative ideas to combat the deadly crisis. NTCA–The Rural Broadband Association represents nearly 850 independent, community-based telecommunications companies and cooperatives and more than 400 other firms that support or provide communications services in the most rural areas of America. This organization's work toward strengthening broadband connectivity in rural areas helps meet two major goals of the battle against opioid misuse: (1) improving access to prevention, treatment, and recovery support services; and (2) strengthening public health data reporting and collection.

THE OPIOID CRISIS HAS A DISPROPORTIONATE IMPACT ON RURAL AREAS

According to the Centers for Disease Control, more than 130 Americans died each day in 2017 from an opioid-related event.[13] The Appalachian and the Southwest regions have been among the hardest hit by the crisis.

These impacts are particularly devastating for rural areas that already face challenging healthcare situations. Rural residents are on average poorer and older than in urban areas.[14] And, although 19.3 percent of the U.S. population resides in rural areas[15], only 10 percent of the Nation's physicians practice in rural America.[16] Additionally, rural areas are home to 70 percent fewer specialists per 100,000 people. Numerous studies, however, have demonstrated both health and economic benefits of telemedicine across multiple medical conditions.[17] These same benefits can be deployed to address the opioid crisis, if sufficient broadband connectivity is achieved.

AVAILABILITY OF SUFFICIENT BROADBAND

Broadband deployment in rural areas is challenged by intensive capital costs that are compounded

by the geographic distance over which facilities must be deployed. Costs must be recovered from a much smaller number of users, as compared to more densely populated urban areas. Moreover, rough terrain in some rural areas can cause additional deployment and maintenance expenses that are not encountered in urban areas.

Small, locally operated providers (such as NTCA members) that operate exclusively in rural markets are driven by a commitment to provide the best service possible to the community in which they serve. According to NTCA survey data, approximately 50 percent of NTCA members provide fiber to nearly 50 percent of their customers, and 60 percent of NTCA members report that nearly 70 percent of their subscribers can receive service at 25 Mbps (megabits per second) or higher. More than 150 NTCA members have been certified as gig capable providers. However, many types of providers serve rural areas, and more than 30 percent of Americans in rural and tribal areas lack access to terrestrial broadband of 25 Mbps/3 Mbps. More connectivity work is required to connect everyone who needs access.

RESPONSES TO THE OPIOID CRISIS RELY ON BROADBAND

Broadband connectivity increases economic activity, enables social interaction, provides educational opportunities, and facilitates better medical care—all critical tools to combat those conditions. Social connectivity enabled by broadband can bridge distance and help to dispel loneliness or feelings of disconnection. Broadband-enabled medical responses include prescription monitoring, e-prescribing, counseling, and prevention campaigns. A rural community with insufficient broadband cannot provide citizens with these benefits, potentially resulting in poorer health outcomes and decreased ability to serve those in recovery from OUD.

Limited employment and educational opportunities in rural areas lead to lower prosperity, and these factors can also contribute to hopelessness that fuels addiction. However, broadband connectivity can spark industrial and entrepreneurial economic development in these remote communities. For example, broadband-enabled distance education supports a much wider range of vocational and career training available on a variety of online platforms, including virtual internships. Dispersed secondary-school students can take coursework from specialized teachers far away. All these experiences allow students to gain the skills necessary to secure better, higher-paying jobs and brighter futures.

EXAMPLE OF HOW BROADBAND CAN ADDRESS HEALTHCARE NEEDS

Critical elements of the programs described below can be transferred to address the opiate crisis by modeling the social, medical, and therapeutic connections enabled by broadband. These measures, in turn, will support critical elements in the prevention of, treatment for, and recovery from SUD.

Hocking County Court

A county court in Appalachia helps clients schedule probation and counseling appointments through videoconferences with court and counseling personnel. These broadband-enabled solutions avoid significant time and costs for clients who live far from town and enable those who may be restarting their employment to avoid missing work. The program also reduces the likelihood of driving under

PROJECT ECHO

Project ECHO is a teleservices model that can help to bridge the gap in delivering substance use disorder services

Accessing specialty healthcare in rural America can be challenging, and patients must often travel many miles to see a provider, especially those in far-flung areas without adequate healthcare facilities or widely available public transportation. Project ECHO® (Extension for Community Healthcare Outcomes) helps meet this challenge by facilitating access to specialty care through rural health front-line providers in a hub-and-spoke model.

Founded in 2003 at the University of New Mexico, Project ECHO® operates more than 130 programs throughout the U.S. today, addressing more than 65 diseases and conditions. Expert specialists at academic hubs connect with rural primary care physicians in local communities through spokes using widely available teleconferencing technology. Primary care providers present patient cases to hub experts and other call participants, cases are discussed, and treatment options are provided. Through these virtual grand rounds, local providers gain access to cutting-edge information, receive support, and refine skills, allowing them to provide their patients with the best possible treatment. To learn more visit the Project ECHO® website at **https://echo.unm.edu/**.

suspension charges for individuals who may drive to appointments without a valid driver's license, and it reduces the incidence of missed appointments where public transportation does not exist.

Virtual Living Room

A broadband-connected workstation in a private room at a county library in McKee, Kentucky enables veterans to access U.S. Department of Veterans Affairs (VA) telehealth and other online services at no charge. This solution supports standard medical appointments as well as mental health and other therapies. The eastern Kentucky site saves veterans an approximately three-hour drive to the VA medical center in Lexington.

Remote Health and Care

An in-home technology pilot in Brandon, Minnesota, connects hospice patients with their loved ones, caregivers, and medical team. In-home hospice patients can view pictures, receive incoming messages, watch medical videos, video chat with family and friends, and listen to music. The system also connects to a variety of wireless activity sensors placed in the patient's home that can alert designated caregivers by phone, email, or text message, and offers the capability for real-time biometric feedback from the system.

ACTION STEPS TO FACILITATE SMALL SCALE BROADBAND PROJECTS

Meeting the opioid crisis will require a diversified, multi-faceted approach, but many effective tools will be powered by rural broadband. Local leaders may consider the following steps to facilitate small-scale broadband projects to increase access to SUD services. They call for local leaders to Identify, Assess, Deploy, Involve, and Communicate:

1. **Identify the prevalence of SUD and illicit substance use in the community**. Prioritize the greatest needs against existing and possible future resources.

2. **Assess broadband resource needs**. Determine what is possible to achieve with respect to prevention, treatment, and recovery; existing broadband availability (e.g. neighborhoods served, maximum broadband speeds, technology available, number of providers); pinpoint where service gaps exist and where new wireless capacity could be used to deliver services to people with SUD; and, identify broadband activities most appropriate to each location.

3. **Deploy the "right" technology and/or service needed to expand access to broadband and match it with the SUD service that is needed**. The potential technology solution will vary depending on the community. Take steps to learn about the technology and service options from broadband providers, equipment suppliers, and other communities about what technology and services they chose and what factors were important.

4. **Involve SUD stakeholders in the community right from the outset**. Stakeholders active in driving services for SUD should be engaged early to work with broadband partners to help develop and guide prevention, treatment, and recovery technology goals and how to implement projects to achieve those goals. They can include medical professionals, educators, economic development officials, faith-based organizations, treatment and recovery professionals, social service organizations, law enforcement and local judiciary, and local broadband providers. Find local champions to take the lead and help make the case for action.

5. **Communicate opportunities to leverage resources and support**. Broadband projects need partners—often from the private sector. Identify potential partners to support broadband small projects. Expanding broadband networks and gaining access to higher broadband speeds does not just happen; it takes a concerted effort. All parties must work together to ensure that the distinct strengths and skills that each party contributes are leveraged effectively.

BRIDGING THE TRANSPORTATION GAP FOR ACCESS TO SUBSTANCE USE DISORDER SERVICES IN RURAL COMMUNITIES

By National Rural Transit Assistance Program

RURAL PUBLIC TRANSPORT

Public transportation in rural communities is often scarce, runs infrequently, and is under resourced. Access to treatment, food, jobs, or education can be improved by working closely with a nearby system's planners to amend a project in an existing regional transportation plan. Bridging the transportation gap to SUD treatment access requires an understanding of the underlying mission of the system. Government transportation systems view human service and medical programs as client-based. Transit is part of a system of mobility services and infrastructure integral to the health of a community and its citizens, especially when public health crises hit the region.

TRANSPORTATION IN RURAL COMMUNITIES

Some rural communities may have limited transportation options, and even those that exist could be difficult to find. State Department of Transportation transit divisions and the National Rural Transit Assistance program can be helpful in identifying regional resources. Often in rural areas the transit will serve several counties or be managed from local community programs or nonprofits. Transit agencies have training and knowledge to manage vehicles and trips. In the absence of a comprehensive transit system, they can help improve mobility for clients.

The Federal Transit Administration provides capital, planning, and operating assistance for rural transit in the Formula Grants for Rural Areas program, which is allocated to rural areas in all fifty States and U.S. territories. Approximately $750 million was provided for this program in FY 2019. In the current environment of rampant opioid addiction, planning must consider transportation needs related to the treatment and recovery from addiction. There are three types of transportation service: demand response, deviated fixed route, and fixed route. These services are managed in Community Action Programs, Area Agencies on Aging, cities, counties, and even hospitals. They serve the general public, and some agencies serve specific programs as well or exclusively.

> "It is important to think about rural transit on a regional level. However, while the actual planning for transit may happen at the State or regional level the implementation is local."

REGIONAL PLANNING

In rural communities, it is important to think about rural transit on a regional level. However, while the actual planning for transit may happen at the State or regional level the implementation is local. In addition, transportation planning and need identification is often separated by years so gaps in the system may have to be addressed in the regional level plans as well as the local transit plan. To improve the system

in your local community you must first identify the programs that service your area.

HOW DO YOU FIND A RIDE?

The below chart provides an example of the process of finding local transit.

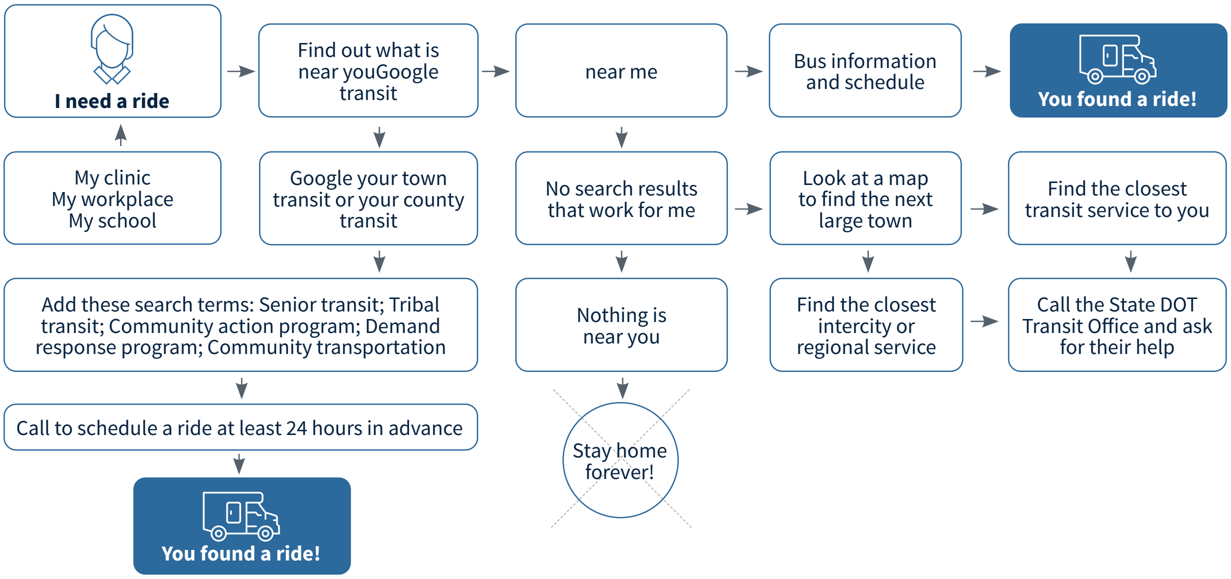

DESIGN MOBILITY SOLUTIONS TO MEET LOCAL NEEDS

Health services often do not know how to assess patient access to transportation. Transportation is usually the second most important priority, after the delivery of the program services. Medicaid agencies may not be aware of every person who needs transportation services. Depending on how the Medicaid program is set up in each State, access to transportation may provide access to medication and other necessary services or only access to treatment.

When designing mobility solutions, assess a client's full transportation needs. Once transit programs are located, schedule time to meet and discuss client needs with the transit agency. They can work jointly to develop a project scope and description for services. Through discussion, identify ways to maximize the value of existing transportation resources to meet local community needs. Determine if the agency has a Coordinated Human Service and Transportation Plan. The planning process assesses resources and needs for seniors and persons with disabilities. The definition of disabilities was recently expanded by the Dept. of Transportation to include persons with SUD. New priorities can be amended into the plan if you work with your transit agency. The below table provides a few examples of local transit strategies.

"Determine if the agency has a Coordinated Human Service and Transportation Plan. The planning process assesses resources and needs for seniors and persons with disabilities (including persons with SUD)."

MODELS AND STRATEGIES TO IMPROVE TRANSPORTATION IN RURAL COMMUNITIES	
**Fixed-route Systems**	Fixed-route bus systems operate on a predetermined route and schedule and are mainly used for general public, commuter (no paratransit), regional connectors, and intercity bus services. These may be managed by public transit agencies, public private partnerships, or private nonprofits, or local and tribal governments.
**Demand-response Transportation**	Demand response transportation (DRT), often referred to as dial-a-ride, are transportation services where individual passengers can request a ride from one specific location to another specific location at a certain time. These services are often used to provide medical access but meet a spectrum of mobility needs including first mile last mile, general public, using ride hailing services, airporters, taxi, taxi voucher, volunteer, and public transit programs.
**Flex-route Transportation Systems**	Flex-route transportation systems or deviated fixed-route systems, feel like fixed route services but can pick up and drop off passengers at locations within a defined distance from the scheduled stops on request. This is a strategy to increase access and usability of existing transit services. DRT can schedule stops so people do not have to call 24 hours in advance or fixed-route services can expand service areas incrementally to reach people who would require DRT to access transit.
**Volunteer Model**	Volunteer transportation programs rely on volunteer drivers. This supports access to medical care, shopping, or nutrition programs. Area Agencies on Aging, hospitals, veteran programs, and other targeted population programs use this strategy to assist program participants. Volunteer reimbursement, if any, does not usually cover full costs of mileage and gas. Some programs trade driving for a voucher for transportation services.
**Coordinated Services Model**	This model involves identifying shared mobility interests between agencies and programs. The goal is to maximize mobility and minimize duplication of services through coordinating and sharing resources, knowledge, and funding. Coordinated services fill gaps in transportation services to efficiently build mobility networks. Key partners for coordinated services include human service agencies, nonprofits, worksites, transit providers, and local or regional economic development agencies.
**Mobility on Demand**	Mobility on demand utilizes technologies, such as smartphones and mobile apps, to increase access to transportation options, increase convenience, simplify payments, and lower costs. Companies like Uber and Lyft, also known as Transportation Network Companies (TNCs), are a form of DRT and sometimes ridesharing.
**Ridesharing Model**	Ridesharing is demand response transportation where a vehicle is shared between one or more persons (vehicle sharing), combining passenger trips with a common destination (carpooling and vanpooling), or using technology to arrange shared rides on short notice or en-route (real-time ridesharing). TNCs have shared ride features in some markets, such as Lyftline and Uberpool.
**Regional Service Model**	Feeder services or regional connector services provide transportation to or from service centers or multimodal transportation hubs. Connector services are implemented to help community members reach medical, educational, and transportation resources. These can be part of a transit system through public or private partnerships or private businesses providing long-distance transportation (i.e., airports or inter-city buses) to specific destinations like health centers or hospitals.
**Mobility Management Model**	Mobility management (MM) helps people connect to transportation options. These programs use trained staff who are knowledgeable about transportation services available regionally. The MM role is to facilitate navigating different transportation modes and options to help riders find and use accessible services.
**Mobility as a Service**	Mobility as a Service is a combination of public and private transportation services within a given regional environment that provides comprehensive, optimal, and person-centered travel options, to enable end-to-end journeys paid for by the user or group of agencies as a single charge, and which aims to achieve key public equity objectives.

ACTION STEPS TO BRIDGE THE TRANSPORTATION GAP

Transportation is a significant challenge for persons with SUD who lack access to public transit or other modes of transportation. Rural communities can implement programs that provide transportation to people on demand, for any reason. Communities that are interested in implementing transportation programs to help address the need should consider the following action steps:

1. **Bring transportation to the table**. Make sure that representatives from the State or local transit office are engaged early. Invite them to join working groups or the taskforce that is formed to address SUD in the community.

2. **Understand the level of transportation that people seeking SUD services need**. Engage in a community prioritization exercise to identify the transportation needs of clients and the areas with the greatest needs.

3. **Put the transportation need on the DOT radar**. Make sure that the transportation need is reflected in the transit plan. Transportation is funded through the U.S. DOT and the Federal Transit Administration only if it is in a plan. If a transportation need is included in the transit plan for the region, that need may be considered for funding.

4. **Identify service gaps and the systems that are available to help**. Identify the closest transit program that can help as well as the clear service gaps. Be sure to address special considerations like disabilities that may impact these needs. Special housing areas for those dealing with SUD will need direct routes to treatment and recovery centers.

5. **Consider whether plans or projects can be adapted to provide support**. Coordinate with the transit program and stakeholders locally or at the State level to develop and amend a project to the State or local Coordinated Human Service and Transportation Plan.

6. **Know the costs and leverage resources**. Work with the local transit to identify the costs for developing the mobility the clients need to be successful. Seek opportunities to leverage resources, engage with other programs, and find public and private stakeholders that share an interest in addressing SUD.

7. **Add access to transportation to the treatment or recovery plan**. SUD programs do not always provide transportation as part of the clinic services. Yet, people seeking substance use services usually lack financial, personal, or physical resources to meet their mobility needs. Find ways to build access to transportation into treatment and recovery modalities.

OVERCOMING ECONOMIC CHALLENGES AMID THE OPIOID CRISIS

By Appalachian Regional Commission

Our Nation's drug crisis is more than a public health emergency. It is increasingly becoming an economic development issue. The harmful impacts of opioid misuse on economic development and the people who make up the workforce cannot be ignored. Local efforts to help build a strong economy—through investments in basic infrastructure, in strengthening entrepreneurship, in expanding transportation options—cannot achieve maximum success if a region does not have a healthy workforce and development options. SUD and opioid misuse drains regional resources, both human and financial, reshapes the fabric of families and communities, slows productivity, and can ultimately make the region less economically competitive.

IMPACT ON THE ECONOMY IN RURAL AREAS

The Appalachian Regional Commission (ARC) understands that to battle the drug crisis, there is a need for a strong response that addresses not only access to treatment and prevention methods, but also the impact on economic development. ARC is an economic development agency that provides Federal assistance to 420 counties in Appalachia for the creation of long-term employment opportunities and economic growth. ARC programs can assist in planning, technical assistance, job training, and the physical infrastructure needed to attract employers and jobs in communities to spur economic growth.

In a study commissioned in 2017 by ARC, the NORC Walsh Center for Rural Health Analysis found Appalachian Region opioid-related overdose mortality rates 49 percent higher than in the non-Appalachian parts of the country (24.6 deaths per 100,000 population ages 15-64 in Appalachia, compared to 16.5 per 100,000 in the non-Appalachian U.S.).[18] Though this study focused on overall mortality rates, age-specific figures are illustrative of the workforce issue facing Appalachia. Among Appalachians aged 25-44, the overdose mortality rate—which includes overdose deaths due to both opioid and non-opioid substances—is more than 70 percent higher than for those living outside the region. Typically, this group includes those in their prime working years, which creates a significant challenge to economic development in rural communities.

There is a clear cyclical relationship between economic challenges and the drug crisis that is visible in Appalachian communities. Some characteristics include an underperforming workforce, challenges of recruiting drug-free workers, higher rates of worker turnover, employee retention, workplace safety, and companies and industries reluctant to do business in the region due to workforce challenges. Anecdotal evidence suggests that sometimes, even in healthy economic environments, companies have to turn away work due to labor shortages, which employers are attributing to substance misuse.

"There is a clear cyclical relationship between economic challenges and the drug crisis that is visible in Appalachian communities."

COLLABORATIVE APPROACHES TO MITIGATE OPIOID CRISIS IMPACTS

Rural communities are adopting collaborative approaches that bring together stakeholders across sectors to respond to the crisis in a more comprehensive manner. ARC, Robert Wood Johnson Foundation, and Foundation for a Healthy Kentucky have jointly released a series of three reports[19] that explore health in the Appalachian Region and ways to improve health outcomes.

First, researchers measured population health in the Appalachian Region and documented disparities between Appalachia and the Nation. The second report describes the methodology used to identify Bright Spots, or Appalachian counties with better-than-expected health outcomes given their characteristics and resources. The third report explores the programs, activities, and initiatives in ten of those Bright Spots through case studies.

Many of the ten Bright Spot counties are taking creative, proactive steps to face the substance misuse issue directly. These efforts include organized substance misuse support groups for addicted individuals and their families, initiatives to curb addictive behaviors, and low-cost disposal sites. Often, these initiatives involve agencies working collaboratively across sectors. These Appalachian communities are addressing substance misuse by working together and dedicating scarce resources to recovery and rehabilitation efforts.

For example, Grant County in West Virginia has launched several initiatives to combat opioid misuse. The county and local organizations are collaborating to form coalitions and offering training in the administration of naloxone, which can rapidly reverse an opioid overdose.

> "These efforts include organized substance misuse support groups for addicted individuals and their families, initiatives to curb addictive behaviors, and low-cost disposal sites."

Their Prevention, Intervention, Treatment, Anti-Stigma, and Recovery (PITAR) coalition is helping coordinate an effort in Grant County that spans all aspects of addressing addiction, from education to mental health counseling to treatment and recovery support services.

The Grant County Health Department has also approved a program to minimize the harmful effects of SUD and is considering providing training in administering naloxone for overdoses. Another regional agency, Potomac Highlands Guild, provides intensive outpatient treatment for SUD. In addition, the Russ Hedrick Recovery Resource Center is a nucleus for recovery activities in the area, serving as a drop-in site to help people with SUDs get into treatment, begin recovery, and find the resources they need for successful recovery.

ACTION STEPS TO DRIVE ECONOMIC DEVELOPMENT

Local leaders looking to mitigate the impact of the crisis on local economic development should:

1. **Utilize data to better understand the extent of the substance use problem on the economy in your community**. Hard data will not adequately capture the devastating impact of the drug crisis on communities, but it is an important starting point that allows for the creation of better-targeted solutions. Communities within the ARC region can refer to the ARC tool (**http://overdosemappingtool.norc.org/**) and communities outside the ARC region should refer to the USDA-NORC Community Assessment Tool (**https://opioidmisusetool.norc.org/**).

2. **Learn more about the (Federal, State, and local) resources available to help combat the opioid crisis**. At the Federal level, the ONDCP-USDA Rural Federal Resource Guide[20] is a one-stop-shop for rural leaders looking for Federal funding and partnership opportunities. It includes information on ARC funds and other partners.

3. **Focus on investment strategies that are regional, strategic, and transformational**. There is no silver bullet to address the opioid crisis, but if a problem is present in a community, the solution is often community-based. In this same spirit, communities should consider taking more of a cross-cutting approach to this multi-faceted problem by bringing together health providers, law enforcement, employers, educators, local officials, and others to work collaboratively and not just in the context of their own organizational silos.

4. **Change the narrative to turn the perceived negative impact into an opportunity**. For many rural areas, the SUD crisis can lead to economic opportunities that include the development of new sectors intended to help people maintain long-term recovery, or the strengthening of existing ones, such as healthcare services.

5. **Support programs that get people with SUD back to work**. For individuals in recovery, a job can be an important component of, and a common reoccurring feature in, successful recovery outcomes. Communities should consider the recovery supports, education, and training needed by individuals in long-term recovery to ultimately get them back into the workforce.

SECTION 3

Prevention

PARTNER INSIGHT AND RECOMMENDED ACTION STEPS

Preventing drug use before it starts is key to building strong and healthy communities. In rural America, communities are often close-knit and have unique strengths to drive coordinated action around prevention. For example, rural leaders can tap into assets like the Rotary Club, cooperative extensions, and local school systems to make big changes with a small investment in messaging around the dangers of substance misuse.

Amid the ever-changing face of drug addiction, rural leaders must look for opportunities to build new community coalitions and broaden existing efforts with additional stakeholders, such as a local hospice care organization. To reach targeted populations, such as youth, farmers, or parents, in a rural area, prevention campaigns that are built around peer-to-peer relationships and tailored messaging can be particularly effective. And, lastly, the growing impact of addiction on rural America has opened doors to engage new partners around prevention, like the philanthropic community.

STOP DRUG USE BEFORE IT STARTS, EARLY PREVENTION AND EARLY INTERVENTION STRATEGIES THAT WORK

By USDA National Institute of Food and Agriculture

EARLY PREVENTION ADVANTAGES

Implementing population-based, preventative, educational programs and resources is a key strategy to achieving positive health outcomes and promoting a positive quality of life in rural America. With respect to effectively addressing SUD, experts have identified prevention as a critical element to curbing the crisis for the future. Evidence-based prevention programs and approaches implemented before substance misuse challenges arise can prevent or delay misuse and abuse by equipping individuals and families with the knowledge and skills to make healthy choices. Prevention and early intervention can also save critical dollars covering treatment and recovery support services by addressing factors leading to substance misuse before it begins.

These prevention-based strategies should be a part of a comprehensive approach for prevention, treatment, and recovery support services in a continuum of care to ensure the best opportunity for effectively addressing substance misuse. A key feature of preventative programs and approaches should be program content and delivery that is based in research and evidence. According to the 2016 Surgeon General's Report on Alcohol, Drugs, and Health, evidenced-based programs can significantly reduce substance misuse and abuse, if implemented well. However, many schools and communities are using programs with the least evidence.[21] Prevention programs should aim to promote protective factors and reduce the impact of risk factors leading to the prevention of opioid misuse and abuse. These programs address substance use, but also promote overall health and well-being.

"A key feature of preventative programs and approaches should be program content and delivery that is based in research and evidence."

Protective factors are strengths that directly decrease the likelihood of substance use and behavioral health problems or reduce the impact of risk factors on behavioral health problems. Examples include strong and positive family bonds, parental engagement, spirituality, and opportunities for meaningful social involvement. Conversely, risk factors increase the likelihood of beginning substance use, of regular and harmful use, and of other behavioral health problems associated with use. Risk factors can include access to prescription drugs, parental separation, childhood trauma, low community cohesion, and permissive attitudes and policies toward substance misuse.

Youth and young adults are important target audiences for prevention efforts as research has shown that early substance misuse, including alcohol misuse, is related to a higher likelihood of developing a SUD later in life. Although, prevention measures have been proven cost-effective, these programs and approaches have reached only about 10 percent of youth across the country.

COOPERATIVE EXTENSION; THE RESOURCE IN YOUR BACKYARD

The Cooperative Extension System provides non-formal education and learning to residents of rural communities by taking knowledge gained through research and education and bringing it directly to the people to create positive change. The service is provided by the State's designated land-grant universities, and there is an extension office in every county and parish in the United States. It is designed to help people use research-based knowledge to improve their lives. The presence of the Cooperative Extension Service enables access to free or low-cost educational measures, such as healthy literacy skills and physical activity and stress management classes, designed to improve mental and physical health as well as reduce pain. Extension professionals have been helping to develop and deliver evidence-based early intervention strategies that can make a difference in addressing SUD.

"The presence of the Cooperative Extension Service enables access to free or low-cost educational measures designed to improve mental and physical health as well as reduce pain."

EVIDENCE-BASED PREVENTION STRATEGIES THAT WORK

Many programs, models, and policies exist that have been proven effective across the lifespan, suggesting that it is never too early or never too late to prevent substance misuse and related challenges. The Cooperative Extension is helping deliver several prevention models in rural communities.

The PROSPER Prevention Delivery System

The PROmoting School-community-university Partnerships to Enhance Resilience (PROSPER) is an innovative prevention delivery system for evidence-based programs. The PROSPER system facilitates sustained, quality delivery of tested and proven programs for sixth and seventh graders that reduce risky youth behaviors, enhance positive youth development, and strengthen families. This delivery system links university-based prevention researchers with established program delivery systems within a State, such as the Cooperative Extension System at the Land Grant University and the public-school system. The model has three components, local community teams, State-level university researchers, and a Prevention Coordinator team in the land grant university, which enables it to address financial and human resource barriers. Ongoing evaluation and technical assistance ensure that programs are implemented as intended, teams continue to perform effectively, and partnership goals are being met. A study conducted by researchers from Penn State University and Iowa State University revealed that children who participated in PROSPER showed lower rates of substance misuse after high school graduation.

PROSPER Prevention Delivery System

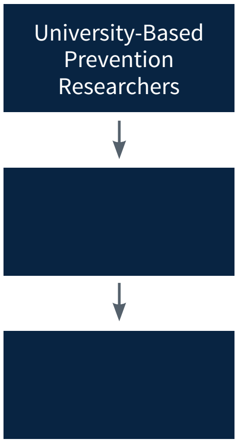

In rural Hawarden, Iowa with a population of 2,200, the community team implemented the PROSPER system to stop illicit substance use in Sioux County. They are using it to deliver two prevention education evidence-based programs—life skills and family skills. Local students study a middle school life skill-building curriculum designed to reduce substance misuse among middle schoolers while the Strengthening Families Program (SFP) is offered to families with sixth graders. SFP is an evidence-based family skills training program found to significantly improve parenting skills and family relationships and reduce substance misuse across adolescence and into young adulthood. The Sioux County PROSPER team is comprised of County Extension, school leaders, substance use prevention professionals, community health providers, ministerial association, and church representatives from the local Youth for Christ Center, law enforcement, and parents.

Chronic Pain Self-Management Program

Finding new ways to manage chronic pain is critical to prevent addiction. The well-regarded model Chronic Pain Self-Management Program (CPSMP) empowers chronic pain sufferers to manage their pain more effectively, reducing or eliminating the need for prescription opioids. Developed in 1996 by Dr. Sandra LeFort, the teaching is what makes this program effective. It gives participants the skills to coordinate all the things needed to manage their health, as well as to help them keep active in their lives. Evaluations of the program found it to be beneficial for participants in terms of coping skills, education, and overall quality of life. CPSMP was developed for people who have a primary or secondary diagnosis of chronic pain. Pain is defined as being chronic or long term when it lasts for longer than three to six months, or beyond the normal healing time of an injury. The University of Arkansas Cooperative Extension partnered with health volunteers, county extension agents, healthcare providers, and pharmacists to implement the CPSMP program and coupled it with an exercise program in four rural counties.

Mental Health First Aid® Education and Training

Communities can also implement capacity-building approaches that promote readiness to address behavioral health challenges that might lead to substance misuse. One such approach is Mental Health First Aid® (MHFA), an evidence-based public education program that encourages early detection and intervention by teaching participants how to identify, understand, and respond to signs of mental illnesses and SUDs, and link them with appropriate professional mental health services and support strategies. It is not a diagnosis and does not replace the need for a professional. Instead it offers concrete tools and answers key questions, like "what do I do?" and "where can someone find help?" The Youth MHFA serves a similar purpose—it reviews typical adolescent development and provides guidance on a range of topics including anxiety, depression, SUDs, disruptive behavior disorders (including ADHD), and eating disorders. It is designed to teach parents, family members, caregivers, teachers, school staff, peers, neighbors, health and human services workers, and other caring citizens.

In Ohio, there are 274 instructors certified to teach MHFA and 188 instructors certified to teach Youth MHFA. Ohio State University Extension plans to offer Youth MHFA to adults assisting youth in several communities to complement other substance misuse prevention programs to build capacity around the State to deal with the opioid crisis.

ACTION STEPS TO IMPLEMENT UNIVERSAL INTERVENTION EDUCATIONAL PROGRAMS

Local and State level systems can also implement universal interventions, which are those that target all members of a given population. In New York, the State health education curriculum now requires content about opioids and heroin as a part of their overall prevention strategy. Implementing this curriculum involves maintaining partnerships with community agencies and bringing resources into classrooms. Rural communities looking to do more in the area of prevention should consider the following:

1. **Make prevention a priority in local community planning efforts**. Prevention should be a deliberate part of a community strategy. Promote viewing challenges through a prevention-focused lens and designate champions for prevention efforts.

2. **Make collaboration key**. Establish multi-sector community coalitions. Equally as important to the prevention of opioid misuse and abuse is the creation and sustainability of effective, innovative, cross sector, supportive partnerships and collaborations including patients, families, educators, health leaders, law enforcement officials, policy makers, and other stakeholders. Community cohesion is an organizing force for bringing about lasting change.

3. **Engage parents and caregivers in community efforts**. Adolescents and young adults misuse prescription opioids more than any other age group, and substance use during the teen years significantly increases the risk of developing addiction. Be sure to encourage and empower parents to engage in proper medication storage and disposal, talks with their teen's doctor, and honest conversations with their teens.

4. **Understand your impact**. As resources allow, include an evaluation of prevention efforts to understand what is working for your community. This information could inform future planning, educate decision makers, demonstrate the value of a program, and improve program effectiveness.

5. **Partner with your local Cooperative Extension team**. Invite Extension agents to participate in community coalitions that focus on the opioid crisis and other healthcare issues, co-host events, partner in content development, and facilitate connections. (Find your local office.)

COMMUNITY DRIVEN AND SHAPED SOLUTIONS CHANGE LIVES

By Community Anti-Drug Coalitions of America

LASTING CHANGE STARTS AT THE LOCAL LEVEL

Community-driven efforts to address the rampant opioid crisis spreading across the nation requires local involvement to develop and shape effective solutions. Rural communities have fewer people, smaller transportation infrastructures, and fewer overall resources to fight the crisis than those available to their urban counterparts. Yet, these rural residents are best equipped to understand the intricacies of their own communities and should be the primary partners in determining the destiny of their own regions when strategies are formulated.

The remote nature of rural communities leads to certain dynamics, such as innovative partnering, inter and extra-regional coordination, and centralized State agencies. External agencies often give direction to local rural communities, but these prescriptive efforts generally fail to take consideration of strong community-level dynamics. These efforts are unable to get any real traction and usually dissipate after external support ends.

"To be effective, efforts to address the consequences of SUD should be tightly coordinated locally."

Stronger and longer-lasting trends are built at the rural community level. These approaches engage community members from the beginning, have a better understanding of the issues important to the rural setting, and effectively leverage available resources and agencies. One of the most prominent examples of community work starting from the ground up are anti-drug coalitions trained by Community Anti-Drug Coalitions of America (CADCA). These coalitions contain three critical components that are necessary for any successful community-driven effort. They include local coordination, the right players at the right time, and actively engaged leadership.

LOCAL COORDINATION

To be effective, efforts to address the consequences of SUD should be tightly coordinated locally. Local coalitions can change the way that communities respond to the threats of SUD by mobilizing the entire community to act. The variance in the backgrounds and development of rural communities has led to several innovations in prevention-related efforts. For example, CADCA coalitions are trained to work with several sectors of a community.

The agents with which rural coalitions work depend greatly on their unique setting. For example, rural coalitions in eastern Kentucky work closely with education and local churches based on a strong reputation in their community, but coalitions in western Pennsylvania work more closely with healthcare agencies because of their growth in that part of the State. Rural coalitions in Utah work closely with a highly centralized State health agency and strong civic organizations. The rise of the use of opioids in that State has also prompted increasing partnerships with parents and healthcare agents. Some of

these examples include coordination across the continuity of care. Rural Kentucky coalitions partner with treatment centers and parent groups to help spread information about the warning signs of opioid use and addictions. There is also a developing understanding of addiction as a disease and of the value of prevention against the cost of treatment.

Rural coalitions that have been in existence for more than three years have often been able to support policy and establish relationships and improve the capacity of their community to anticipate problems. A rural Tennessee coalition coordinated efforts with a State public health policy initiative to reduce smoking through public building smoking bans. A State Quitline promotion in coordination with the Parks and Recreation department was leveraged for a campaign against increased vaping and electronic cigarette use in public areas.

THE RIGHT PLAYERS AT THE RIGHT TIME

CADCA community prevention coalitions are trained and positioned to plan and implement their work alongside policy initiatives using recently available data. Rural coalitions work by assessing the community and the issues of greatest concern, then identifying a capacity for strengthening old bonds and starting new partnerships before formulating a strategic direction. The information they gather often becomes a valuable community resource. A rural Georgia coalition was able to create a directory of resources that soon replaced an out-of-date State instrument.

Coalitions should consist of a minimum of 12 community sectors participating in their group, including but not limited to youth under age 18, parents, businesses, media, schools, youth-serving organizations, law enforcement agencies, religious or fraternal organizations, civic and volunteer groups, healthcare professionals, State or local or tribal agencies with expertise in the field of substance misuse, and other relevant organizations involved in addressing illicit substance use in the community.

ACTIVELY ENGAGED LEADERSHIP

An actively engaged leadership and action by stakeholders within rural communities on any taskforce or coalition are necessary components for success in addressing substance use and its consequences. For example, a CADCA coalition is well connected to most parts of its community by design and knows the strongest sectors with whom to work and those persons in leadership positions. The coalition acts as a convener, the agent by which sectors who would not normally interact can create lasting partnerships. As conveners, they provide a forum for sector leadership to become engaged in prevention, treatment, and recovery efforts.

"An actively engaged leadership and action by stakeholders within rural communities on any taskforce or coalition are necessary components for success."

ACTION STEPS TO CREATE COMMUNITY COALITIONS

Many States and local jurisdictions have implemented successful and innovative working groups, a drug focused taskforce, or a coalition to handle the opioid crisis in their communities. Four ways that local leaders can get started include:

1. **Identify areas of concern that could be addressed by community-level prevention, treatment, and recovery efforts**. Wherever possible, add data to help tell the story.
2. **Establish connections with community, county, region, and State-level stakeholders**. Rural communities have a natural partner in county and State agencies.
3. **Identify and connect with the critical stakeholders in the community affected by SUD such as business and healthcare related leaders**. Schedule regular meetings to start building an action plan.
4. **Seek opportunities to learn more about and connect with relevant organizations to learn about coalition building and training that could provide continued support.**

TAKING ACTION TO ADDRESS SUBSTANCE USE DISORDER IN THE FARMING COMMUNITY

By American Farm Bureau Federation / National Farmers Union

OPIOID IMPACTS ON FARMING COMMUNITY

A survey by Morning Consult commissioned in 2017 by the American Farm Bureau Federation (AFBF) and National Farmers Union (NFU) revealed disturbing trends.[22] OUD is deeply affecting the farmer's fields. Three-in-four farmers and farm workers (74 percent) are or have been directly impacted by illicit opioid use, either by knowing someone with SUD, having a family member with SUD, taking an illegal opioid, or dealing with addiction personally. Forty-five percent of rural adults have been directly impacted, having experienced one of the above factors. Three-in-four farmers (77 percent), as well as those who work in agriculture generally, say it would be easy for someone in their community to access opioids illegally.

These are worrisome results, not just because of their frequency, but also because of the difficulties farmers face on a regular basis. Farmers are often short-handed and find it difficult to take time off when they or a family member needs treatment. Healthcare providers and facilities are in short supply and often far away, creating multiple barriers to positive health outcomes for farmers in need of care.

> "OUD is deeply affecting the farmer's fields. Three-in-four farmers and farm workers (74 percent) are or have been directly impacted by illicit opioid use, either by knowing someone addicted, having an addicted family member, taking an illegal opioid, or dealing with addiction personally."

THE FARMER'S ROLE IN FIGHTING OPIOID MISUSE

Farmers trust farmers. Often operating in isolation, they depend on each other for help. They share the experience of battling the elements to care for land and animals that sometimes resist their best efforts. Farmers, then, are their own best hope. Since they understand one another in ways outsiders do not and cannot, they are in a unique position to come alongside and help others in the farming community.

The American Farm Bureau Federation and National Farmers Union launched the Farm Town Strong awareness and education campaign in 2018 based on a longstanding precedent of farmers helping farmers. Both organizations had been looking for ways to work together to address this crisis, and Ohio Farm Bureau had made opioids a major policy initiative prior to the campaign, lobbying the State legislature to address opioid addiction.

State organizations responded to the national call to action from AFBF President Zippy Duvall and NFU President Roger Johnson, who worked closely with other farm organizations to help spread the word. They

hosted each other at their organizations' annual conventions and shared expert opinions in a program about the rural opioid crisis on RFD-TV. The Pennsylvania, Ohio, and Utah Farm Bureaus, as well as Utah Farmers Union, participated in USDA roundtables on the issue. In addition, State and national affiliates of Farm Bureau and Farmers Union endorsed the efforts of an anti-addiction program developed in Des Moines, Iowa, to train farmers to recognize and combat signs of addiction in their communities.

FarmTownStrong.org, a website jointly developed by AFBF and NFU, raised awareness in farm media and farm communities. It provides farmers, ranchers, rural Americans and others with easy-to-find resources for treatment, crisis intervention, drug disposal, and prevention. The website features links to behavioral health treatment, buprenorphine for physicians, and treatment and MAT programs. There are also links to support services for families and children, prevention resources, and information about naloxone to prevent death from opioid overdose. The site also features a drug disposal locator tool, information on home drug disposal and the National Prescription Drug Take Back Day.

HOW THE FARMING COMMUNITY CAN HELP ITS OWN

FarmTownStrong.org is only a first step towards tackling the scourge of addiction. The problem is much, much bigger and more complex than anything one group can handle alone, but America's farmers and ranchers can help in their own unique ways. Farm Bureau and Farmers Union are grassroots organizations committed to serving their members' needs and providing forums for meeting and discussing key issues of importance to farmers, like the opioid crisis. State and county affiliates are discussing the issues raised at these forums and how to effectively address them. Committees at AFBF and NFU are asking what more they can do and are encouraging members to get involved.

Justice Louis Brandeis once observed that States are "laboratories of democracy," places where free people can try new ideas on a small scale before suggesting they might work nationally. Farmers in rural America are working hard in the lab right now.

FUTURE FARMERS OF AMERICA

CAMP MUSKINGUM

The Future Farmers of America (FFA) is a national organization dedicated to preparing members for leadership and careers in the science, business, and technology of agriculture. Camp Muskingum, located in Carroll County, offers camp sessions for all Ohio FFA members. At a recent session in 2018, campers were polled about their experience and opinion regarding drug use. According to the poll of 961 people, 392 said their parents never talked to them about substance use and 330 said their parents only talk about it once per month. Students were also asked about their participation in 4-H, FFA and Farm Bureau and whether they felt those activities helped deter them from substance abuse. Nearly 364 said that belonging to those groups "definitely" made a difference and 299 said that it "probably" does. This provided an opportunity to have an open discussion about drug use and prevention with youth during the camp sessions.

ACTION STEPS THAT FARMERS CAN USE TO HELP ADDRESS ILLICIT OPIOID USE

There are many ways the farming community can help, such as sitting down with family members to talk about the deadly danger posed by SUD and opioid addiction, and how everyone can work together to avoid it. Other ways include:

1. **Volunteer to help persons seeking treatment or in recovery** and participate in initiatives to help, e.g., Drug Take Back programs.
2. **Advocate for better insurance coverage** for addiction treatment and recovery support resources.
3. **Invest in training and educating the farm community** about SUD, opioid misuse, and the Federal, State, and local resources that are available to help.
4. **Work with State, regional, and local governments**, banding together to apply for grants that will bring better services home.
5. **Tap resources unique to the farming community**, such as 4-H clubs, to help drive prevention and awareness-raising activities within the local community.

HELPING STUDENTS DEVELOP INNOVATIVE SOLUTIONS TO THE OPIOID CRISIS

Successful prevention efforts take innovation and partnership. With the breadth of their impact on a rural community, school systems can play an important role not only in transforming a community narrative around substance use but also in recreating a sense of hope and purpose for the future. One example of such effort is the Kentucky Valley Educational Cooperative.

The Kentucky Valley Education Cooperative (KVEC) contains 22 rural public school districts that border West Virginia, Virginia, and Tennessee, which combine to create a geographic area larger than Connecticut. This region serves more than 50,000 students and more than 3,000 educators. The mission is to reframe the education and the community narrative in Appalachian Kentucky by building the human capital of educators, learners, and community members to work together to solve pervasive challenges. KVEC invests in people and not programs and embraces a transformative learning model, which is learning that engages the participant in connecting passion, purpose, and pathway to create real world solutions to pervasive challenges. KVEC initiatives include Building it Forward and the Forging Innovation in Rural Education (FIRE) Summits.

Building It Forward: Hands-on Approach to Learning

The cooperative funded three eastern Kentucky vocational schools to design and build tiny movable homes in 2016. The "Building It Forward" project gives students experience with a wide range of construction skills, such as plumbing, wiring, carpentry, design, budgeting, heating, and cooling. This strategy ensures the cooperative's schools offer classes that are interesting and valuable to students, and provides them with marketable skills, whether or not they plan to attend college. Each school involved receives a base amount of $15,000 and the students build the homes from August until April. Each house is equipped with air conditioning along with a kitchen, bathroom, and bedroom area. The program is designed to be financially self-sustaining. Eighty percent of the amount above the final auction bid goes to each school to build a new house the following school year.

Fire Summits Fosters Youth Innovation and Local Response to the Opioid Crisis

The FIRE Summits serve as a catalyst for energizing and accelerating strategies to improve the quality of education for learners everywhere. Drawing on resources made available through a U.S. Department of Education Race to the Top Grant, the twice-yearly event strategically drives innovation in the classroom by focusing on connecting students to their passions to reach goals and achieve a larger purpose. In recent years, students have addressed the opioid crisis in Kentucky through a series of projects, including:

- **Protecting First Responders to the Opioid Crisis**. National winner of the Samsung Solve for Tomorrow 2018 competition, this project was developed by a student from Ashland Middle School. The students worked with local first responders, police officers, and addiction centers to develop a proprietary device that allows first responders to safely collect hazardous needles left behind by opioid and other drug users. They also used coding skills to develop an online database that local community members can access to learn more about the opioid crisis and report hazardous needles found in public areas.
- **The Empty Chair Project**. Winner of the Appalachian Regional Commission Humanitarian Award for 2018, this project was developed by students from Pikeville Middle and High Schools. Students built an app that helps people learn signs and symptoms of opioid misuse and find opportunities to get help with addiction. A significant community partnership engaged a broad cross-section of partners to develop the solution.
- **YMO "Yours, Mine, and Ours" Stories**. Magoffin County High School Students developed a program to raise community awareness about the personal side of opioid abuse. They began collecting and publishing stories submitted by a quarter of the student population on personal experiences with the negative impacts of opioid abuse. A companion element of the program involved community awareness, supports, and resources for treatment. YMO Stories won the Appalachian Renaissance Horizon Award for 2018.
- **P.U.R.E. KY—Providing Ultimate Recovery in Eastern Kentucky**. Paintsville High School students developed a comprehensive initiative aimed at elevating community awareness to the opioid crisis through multiple communication channels that included community events, publications, focus groups, and community surveys.

To learn more, visit **www.theholler.org/**.

SECTION 4

Treatment

PARTNER INSIGHT AND RECOMMENDED ACTION STEPS

The need for greater access to treatment in rural America is vast. Healthcare providers, practitioners, and funders face enormous challenges in delivering treatment services in rural communities because of geographic distance, limited medical facilities and healthcare workforce, and gaps in infrastructure. Beyond access to treatment, there is also a continuous need to improve the quality of existing care in rural America and to break down the barrier of stigma that may keep a person living in a rural place from accepting treatment even if it is available.

To meet these needs, rural leaders must explore new models for increasing access to treatment, grow the healthcare workforce, develop infrastructure and capacity to use technology for remote service delivery, and educate community members about treating addiction as a disease to encourage community support. With innovation and partnerships to better leverage existing services and programs, local leaders can ensure that people in their community get the help they need to get clean and stay healthy.

STRENGTHENING THE RURAL HEALTHCARE NETWORK FOR PERSONS SEEKING TREATMENT

By National Rural Health Association

RURAL IMPACT OF THE USE OF ILLICIT SUBSTANCES AND PRESCRIPTION OPIOID MISUSE

The opioid crisis spreading across the United States has widened the already growing rural-urban healthcare gap. Effective, evidence-based treatment of SUD or OUD is urgently needed in rural communities. However, multiple barriers prevent appropriate treatment availability and quality, including decaying rural mental health treatment infrastructure, lack of regional coordination of treatment resources, inadequate support for rural physicians, and shortages of physicians who can and are willing to provide MAT for treating OUD. This approach combines behavioral therapy with one of three medications approved by the FDA for treating OUD—methadone, buprenorphine, or naltrexone.

To more successfully treat OUD in rural communities, public and private sector leaders must improve access to treatment, expand the healthcare workforce, and provide more customized treatment to the needs of individual rural communities.

STATE OF RURAL HEALTHCARE

Currently rural America lacks the necessary healthcare infrastructure to effectively treat the consequences of prescription opioid misuse and the use of illicit substances. Rural providers are a critical part of their communities and often serve as the backbone of the rural economy. Yet, 118 rural hospitals have closed since 2010[23], and due to financial strains nearly 700 more rural hospitals are financially vulnerable and at high risk of closure. If they close, rural patients would need to seek alternatives for 11.7 million hospitals visits, 99,000 healthcare workers would face unemployment, and $277 billion in GDP would be lost.[24]

> "Rural providers are a critical part of their communities and often serve as the backbone of the rural economy."

Behavioral healthcare options in rural communities have become alarmingly scarce. As of 2018, 13 percent of rural counties employ no behavioral health providers. Nationally, 65 percent of rural counties do not have a psychiatrist, 47 percent lack a psychologist, and 81 percent lack a psychiatric nurse practitioner.[25] Of the 2.2 percent of physicians who have obtained a waiver to prescribe MAT, less than 10 percent practice in rural counties.[26]

IMPROVING ACCESS TO TREATMENT AND RECOVERY SUPPORT SERVICES

Research has shown MAT is the most effective therapy to treat OUD. Primary care providers may be more likely to implement MAT into their practices with greater accessibility to relevant training, community

"hub and spoke" programs or telehealth models, such as the successful Project ECHO, and enhancing care coordination and management. Regulatory and community barriers to MAT can be resolved by easing unnecessarily restrictive licensing standards, enhancing payment for MAT by State Medicaid programs, and educating community members to reduce opposition to MAT.

For example, Seaport Community Health Center in Belfast, Maine provides integrated opioid and substance use treatment in a FQHC that includes counseling, a buprenorphine treatment program, group counseling, and psychiatry services. Patients must agree to participate in an intensive outpatient program sponsored by a local community mental health center and remain drug-free for the duration of treatment.

Additionally, the expansion of team-based and integrated models of substance use, mental health, and primary care to maximize scarce resources will help streamline OUD treatment. To ensure that rural residents can access services, community-based solutions are needed to develop programs to assist individuals, including transportation, childcare, and housing.

PROVIDING SUPPORT FOR RECOVERY

Opportunities for community involvement and connections also bear a strong impact against addiction challenges. Programs targeted at reducing stigma, providing supportive housing, offering vocational training, and providing safe opportunities for social and recreational engagement are critical to fight opioid misuse.

- The **Vermont Recovery Network**, funded by the Vermont State Legislature, is a statewide network of peer-recovery centers that help people find and maintain recovery, prevent relapse, and return to recovery if relapse occurs. They are local, consumer-driven, nonresidential facilities providing peer support, sober recreation activities, volunteer opportunities, and community education.
- **Project Lazarus** organizes community resources to strategically reduce opioid use in all North Carolina counties as well as across the country. These include Project Bald Eagle in Pennsylvania, Winnebago County Heroin Task Force in Wisconsin, Clark County Collaborative in Ohio, and Washtenaw Health Initiative Opioid Project in Michigan. This community-based public health project aims to build public awareness of substance use through

> "Programs targeted at reducing stigma, providing supportive housing, offering vocational training, and providing safe opportunities for social and recreational engagement are critical to fight opioid misuse."

broad-based educational efforts and establish coalitions to engage a broad range of community providers, agencies, and organizations. By identifying data needs for planning and evaluation to build awareness, Project Lazarus customizes programs to local needs, tracks progress, and sustains support and funding. Communities are urged to start “where they are” and to identify evidence-based prevention initiatives to address specific local needs.

- **Project Vision**, also based in Vermont, is a locally organized collaboration between law enforcement, healthcare providers, schools, government, and community agencies with goals to empower the community and strengthen their futures. Three committees (Crime/Safety, Substance Abuse, and Community/Neighborhoods/Housing) work together to implement a drug market intervention model and community engagement to reduce the overall supply of opioids (heroin and illicitly distributed prescription opioids) in rural Vermont.

EXPANDING THE SUBSTANCE USE WORKFORCE

To encourage more providers to practice in rural areas, especially those with SUD training, programs must focus on the challenges of providing care in these communities. Those who grow up in rural areas and choose to practice in those areas later are more likely to continue providing care in rural communities throughout their careers. Efforts to encourage healthcare professionals to practice in rural communities should include the recruitment of rurally based students interested in pursuing healthcare careers and, ideally, specializing in SUDs.

EMBRACING NEW HEALTHCARE DELIVERY MODELS

Telehealth services allow expanded care options for communities that cannot yet support a larger workforce to address SUDs, while still retaining a network of local healthcare providers. Patients in far-flung areas can meet with specialists without the regular burden and expense of travel, increasing the likelihood of continued adherence to treatment. Research has shown successful treatment outcomes, improved patient satisfaction, and cost savings.

The University of Washington School of Medicine’s “telepain” program focuses on pain management to reduce unnecessary opioid prescriptions. Project ROAM (Rural Opioid Addiction Management) supported rural providers in Montana, Washington, Wyoming, Alaska, and Idaho who are treating patients using buprenorphine. The model was successful in supporting rural providers but was difficult to sustain.

“Telehealth services allow expanded care options for communities that cannot yet support a larger workforce to address SUDs, while still retaining a network of local healthcare providers.”

ACTION STEPS TO SUPPORT CARE NETWORKS

Positive patient health outcome objectives are more likely reached in rural communities with expanded access to treatment and recovery support services. This approach requires rural leaders to:

1. **Build a sustainable continuum of outpatient and inpatient services**. This should include MAT, psychosocial treatment (*e.g.,* structured counseling, motivational enhancement, case management, care-coordination, psychotherapy, and relapse prevention), mental health services, community involvement in recovery, and integrated primary, substance use, and mental health services.

2. **Expand access to telemedicine services to treat SUD**. Innovative care delivery models enabled by technologies, such as telehealth, telecounseling, video conferencing, remote monitoring, and diagnostic scanning, are an important way to bridge the gap in healthcare access.

3. **Invest in alternative treatment delivery methods**. Explore models, such as mobile clinics, school, and workplace-based health programs, and home visiting programs. The effectiveness of telemedicine is reliant on the speed and quality of broadband. Mobile clinics can visit community sites to deliver services and can help people who lack access to specific healthcare services or would otherwise have to travel long distances to see a provider.

4. **Increase the number of healthcare professionals practicing in rural areas**. Expand their capabilities, especially in the areas of substance misuse. Form partnerships with local and State advance practitioner training programs, medical, physical therapy, and pharmacy schools that help develop and maintain the workforce residencies in rural areas. Engage local young people in the healthcare industry. Use innovative strategies to educate and train those who desire to work and live in a rural community.

5. **Encourage and support peer-to-peer community programs**. These should seek to break down stigma and support communities during recovery.

UNDERSTANDING WHY MEDICATION ASSISTED TREATMENT IS DIFFERENT IN RURAL COMMUNITIES

By The Pew Charitable Trust

MEDICATION-ASSISTED TREATMENT FOR OUD

MAT is the most effective therapy for OUD. Multiple studies have shown that people with OUD who receive MAT are less likely to die of overdose, use illicit opioids, or contract infectious diseases that can be spread through injection drug use, such as HIV and hepatitis C, than people who do not.[27] Additionally, patients on MAT remain in therapy longer than people who receive other treatment.[28]

MAT for OUD combines behavioral therapy with one of three medications approved by the FDA—methadone, buprenorphine, or naltrexone. These medications work to relieve the symptoms of opioid withdrawal or block the effects of opioids, while behavioral therapies help patients improve coping skills and reduce the likelihood of relapse.[29] MAT can be delivered in outpatient settings, such as a physician's office, or inpatient facilities, such as a hospital or residential facility. Although some patients may require inpatient services, outpatient settings provide an appropriate level of care for many patients.

> "MAT is the most effective therapy for OUD. Multiple studies have shown that people with OUD who receive MAT are less likely to die of overdose, use illicit opioids, or contract infectious diseases."

The medications approved by the FDA for OUD treatment have different mechanisms of action, which is the specific biochemical interaction through which a drug substance produces its pharmacological effect, and these medications are regulated by States and the Federal Government differently. As with other chronic brain disorders, the optimal choice of medication varies from patient to patient.

Methadone for OUD is available only at OTPs, which are State and Federally regulated facilities that patients visit, usually daily, to take MAT medications under the supervision of staff and receive counseling and other care services. Buprenorphine and naltrexone are available at some OTPs and in other settings, such as primary care practices and community hospitals. Physicians, nurse practitioners, and physician assistants must complete training to obtain a Federal waiver to prescribe buprenorphine. Naltrexone is available from any practitioner who is authorized to prescribe medications and can be delivered through a monthly injection.

ACCESS TO MAT IN RURAL COMMUNITIES

People with OUD living in rural communities often face barriers to accessing medication-assisted treatment, including fewer transportation and treatment options compared with those available in urban areas, according to a study conducted by Maine Rural Health Research Center.[30] For instance, patients in low or moderately populated areas must usually travel farther to access OTPs.[31] Office-based treatment options are particularly limited in rural communities—one study found that 29.8 percent of

rural Americans live in a county without a buprenorphine provider, compared with only 2.2 percent of urban Americans.[32] A survey of rural physicians published in the Annals of Family Medicine in 2017 found that worries about diversion (the redirection of drugs from legal, medically authorized uses to illegal uses), constraints on their time, and lack of access to mental health supports for their patients were the top barriers to incorporating buprenorphine into their practice.[33]

Moreover, SUD treatment centers in rural areas often do not offer the same scope of services as their urban counterparts. Rural facilities are less likely to provide buprenorphine; they also offer fewer wraparound services, such as case management and recovery groups that have been shown to improve health outcomes. These facilities also rely more heavily on public funds to provide treatment than treatment centers in urban areas do, according to a recent assessment of treatment centers.[34] Resource limitations can contribute to decreased availability of evidence-based treatment, with fewer tailored options and specialized providers to treat patients with complex needs.

PROMISING STRATEGIES FOR RURAL COMMUNITIES

Any comprehensive approach to deliver treatment must include education on MAT for OUD, psychosocial services, care integration, and education to providers.[35] The following strategies incorporate these components and show promise in increasing access to treatment in rural communities:

Care Coordination

Care coordination facilitates the delivery of medication and behavioral health services associated with MAT. Research from the Agency for Healthcare Research and Quality shows that system reforms that emphasize care coordination can address some of the barriers to accessing MAT in rural areas. For example, in 2013, Vermont pioneered "hub-and-spoke" systems in which patients start treatment and receive a higher level of services at OTPs (hubs). Once patients are stabilized (i.e., misuse has reduced or stopped, and cravings are diminished), patients are transferred to long-term management with community-based providers (spokes). Care coordination between the hubs and spokes, which aims to address the patient's evolving treatment needs, is an essential part of the model. A report in the Journal of Addiction Medicine found that Vermont's treatment network resulted in the State having the highest per capita capacity to treat OUD patients in the country: 10.56 people in treatment per 1,000. Vermont also saw a 64 percent increase in physicians authorized to prescribe buprenorphine.

> "Any comprehensive approach to deliver treatment must include education on MAT for OUD, psychosocial services, care integration, and education to providers."

Care Integration

Integration of mental and physical healthcare brings together components of care delivery to improve health outcomes, relying heavily on collaboration among mental health and other healthcare professionals, according to the SAMHSA and The National Council for Behavioral Health. For example, a FQHC in rural northern Michigan implemented an integrated approach to deliver MAT to its OUD patients, an undertaking documented by the Rural Health Information Hub. With funding from the HRSA and the SAMHSA, the FQHC established a partnership with a local clinical practice and a behavioral health organization. FQHC patients are screened

for OUD and referred to on-site providers who are waivered to prescribe buprenorphine and provide behavioral health counseling. A formal evaluation has not been completed as of 2018, but preliminary evaluation results indicate that 32 percent of patients remained in treatment for 18 months or more, which is comparable to reported outpatient treatment retention rates.

Telehealth

Rural health systems with sufficient technological capabilities may consider implementing promising practices, such as distance learning and telemedicine. For instance, Project ECHO—which launched in 2003 with the support of Federal, State, and private funds—connects primary care providers with a team of specialists of varying disciplines who practice elsewhere using video-conferencing technology. The specialists provide guidance on treating an array of diseases, including OUD, and consult on challenging patient cases. After starting an ECHO program in 2005, New Mexico increased the per capita number of providers authorized to prescribe buprenorphine, including those practicing in underserved areas. Moreover, an analysis published in The Journal of Addiction Medicine of West Virginia's Comprehensive Opioid Addiction Treatment program, a telemedicine model that uses video conferencing for buprenorphine prescribing and medication management, found that outcomes, such as abstinence from drug use, were comparable to those who receive MAT in person.

ACTION STEPS TO ADOPT INNOVATIVE TREATMENT MODELS

Many States and local jurisdictions have implemented innovative treatment models that have shown promise in saving lives by connecting patients to MAT. Local leaders can help advance these initiatives and access to MAT by taking action to:

1. **Facilitate** the establishment of treatment in specialty and primary care settings in the community or region.
2. **Educate** the local community and leadership on the value of MAT.
3. **Assess and leverage** and leveraging local and regional resources to implement programs.
4. **Adapt** treatment delivery models to address community-specific needs, including needed provider training.
5. **Support** State leaders in their efforts to address the opioid crisis.

MOVING FROM LOCK'EM UP TO PROVIDING TREATMENT AND SUPPORT

By National Sheriffs' Association

In rural communities, jails have become a revolving door for individuals struggling with mental health and SUD. More than 10 million people, at least half with SUDs, are incarcerated around the country annually. People suffering with mental health and SUD come in and out of jail with arrests and incarceration. Without effective intervention before they are released, the disorder typically continues, and the cycle begins upon release often with a dramatic crime rate increase, while those who are most vulnerable, remain sick.

"In rural communities, jails have become a revolving door for individuals struggling with mental health and SUD."

In some cases, incarcerated persons with SUD face fewer barriers to treatment access, like the absence of negative social networks and family obligations. There is no need for health insurance. Medical care and treatment beds are always available, and inmates can immediately see their treatment specialists and caseworkers without the need to secure transportation. The opportunity for freedom from SUD is greatest within this controlled environment.

Jails are in a unique position to initiate treatment to individuals struggling with SUDs and withdrawal in a controlled, safe environment. To achieve optimum outcomes with jail-based MAT, leaders must look beyond basic law enforcement tactics to create a comprehensive and strategic approach with local communities, State, Tribal and Federal government. The present substance use problem underscores the need for law enforcement to take a more active and compassionate role in addiction treatment.

ADOPTION OF MAT IN PRISONS

Traditional approaches to public safety, including, the threat of arrest and incarceration, are not enough to deter a person dealing with a substance use problem. The constant, overwhelming need to fuel the addiction is often too great for standard deterrents to prevent property crime. As a result, law enforcement officers have had to learn how to adjust their approach to law by incorporating MAT treatment into jail-based programs while persons with a SUD are in custody.

Over the past few years, MAT programs in jails have rapidly expanded into 30 States. Now sheriffs and jail administrators are working to collaborate with support services in the community to ensure follow-through treatment after release of inmates in recovery. The upcoming "Jail-Based Medication Assisted Treatment: Promising Practices, Guidelines, and Resources" handbook—from The National Security Agency, the National Commission on Correctional Healthcare, and the National Institute of Corrections—is a valuable new resource for rural, suburban, and urban sheriffs and jail administrators

seeking innovative evidence-based drug treatment.

Jails are the intersection where public health and public safety crises collide. While a jail sentence may seem like rock bottom for those convicted, this time can also serve as a window of opportunity to address factors that led to incarceration. Though the corrections environment was not designed for addressing substance use problems, law enforcement officials can serve a vital public health need by providing incarcerated individuals with treatment and tools. This reduces the risk of fatal overdoses and addresses the underlying cause of criminal activity that poses a threat to public safety.

The Massachusetts MATADOR (Medication Assisted Treatment and Directed Opioid Recovery) statewide pilot program provides three types of medication-assisted treatment in five county correctional facilities. The Middlesex County Sheriff's Office (MCSO) serves rural, suburban, and urban communities and adopted and implemented a successful data-driven model program.

MIDDLESEX COUNTY MATADOR PROGRAM SPOTLIGHT

About 80 percent of individuals sent to the Middlesex County Jail have a SUD, and half of them suffer severe addiction requiring immediate medical detoxification. In 2017 the correctional facility's medical unit counted 178,000 contacts with patients, far outpacing the largest hospital emergency room in the county. The Massachusetts Department of Public Health's Section 55 Opioid Overdose Report found that individuals released from jails or prisons are 120 times more likely to die of an opioid-related overdose than the rest of the adult population.

In response Middlesex launched the MATADOR program to treat substance use with naltrexone. As part of the program, they receive health assessments, detox treatment, monitoring, regular medical attention, and work with a recovery coach or "navigator" during incarceration. Upon release, the MATADOR navigators continue to serve as advocates. They ensure patients have a reentry plan in place, are scheduling and attending medical and program appointments, and have support during setbacks. The navigator also helps ensure clear and timely communication between community healthcare providers, support program locations, drug courts, and the sheriff's office.

MATADOR program participants are deemed successful after six months when they are well into their reintegration back into the community, have established routines, are familiar with the network of healthcare and other resources available to them, and their continuity of care has been established. Navigators work closely with research staff at the MCSO to regularly identify and track the progress of each enrolled participant, and research staff communicate with each healthcare provider to ensure program compliance as well as any interaction with law enforcement.

Three years after inception, research showed that by applying a data-driven public health approach to those with OUD within correctional custody, the MATADOR program is both saving lives and reducing future incidents of crime. For example, 96.2 percent of MATADOR participants (regardless of their success or failure in the program) had not succumbed to a fatal opioid-related overdose post release as of June 2018.

ACTION STEPS TO START A JAIL-BASED MAT PROGRAM

Challenges like slim budgets and limited staff assistance continue to hinder rural communities from implementing comprehensive jail-based MAT programs. Most sheriffs, particularly those in the western United States, serve vast areas with little or no community services that can provide continuity of care after a person is released from a detention facility. Incarceration is a window of opportunity for individuals that may have spent years, even decades, using opioids, to come clean for the first time. People should not have to come to jail to get good treatment, but while there, it should be viewed as an opportunity. Enforcement professionals in rural communities should consider the following steps:

1. **Learn about a variety of jail-based MAT programs**. There is no one-size-fits all jail-based MAT model. Identifying different options will help law enforcement professionals identify a model that could work or tailor one to a specific vision.

2. **Identify and recruit like-minded correctional professionals to start**. Correctional professionals may not be comfortable with this process at first. Initiate a pilot with a team of like-minded colleagues, working with a smaller group to start (e.g., juveniles or women) for a specified period of time. This will allow time to find, assess, and fix any issues before a wider roll out of the program.

3. **Determine what resources you have and what resources you need**. Take stock of the resources needed to introduce a jail-based MAT program. Assess what is readily available and what resources need to be acquired before a program can be implemented.

4. **Leverage the resources of community partners**. A jail treatment program relies not just on the services within the system but also on services and programs delivered by community partners.

5. **Encourage enforcement professional peer-to-peer learning opportunities**. Host workshops and engage with other rural law enforcement and correctional professionals dealing with similar situations in other counties or States. Increasing opportunities for direct exchanges with peers will help increase the level of comfortability with these initiatives and dispel myths.

6. **Collect data**. Data collection can be daunting for some, but it should not be feared. Collecting baseline data that includes demographics and basic performance measures is essential. Programs like MATADOR may not work for every individual, but the potential positive outcomes regarding harm reduction should not be overlooked.

MAKING DRUG COURTS WORK FOR RURAL AMERICA

By Center for Court Innovation

Drug courts rank among the most successful criminal justice innovations in recent decades, gaining widespread support in States across the country. More than 3,000 drug courts exist, including DWI courts, mental health courts, and veteran treatment courts.[36] Using a collaborative, team-based approach, drug courts combine court-supervised treatment with an array of supportive services, ongoing judicial monitoring, and motivational incentives and sanctions. These serve as an alternative to conventional case processing and jail.

In addition, large-scale studies like the Federally funded Multi-Site Drug Court Evaluation have helped to identify specific, evidence-based practices that promote drug court effectiveness. This research, in turn, has been distilled into the Adult Drug Court Best Practice Standards, a detailed set of concrete practices that all drug courts should strive to achieve.

RURAL DRUG COURTS FACE SPECIAL CHALLENGES

Drug courts in rural jurisdictions have a few challenges. Drug courts combine help, in the form of treatment and other services, with accountability, such as randomized drug testing and utilize principles of behavior modification, to help guide participants toward long-term success. To promote abstinence and detect relapse, it is critically important that drug courts engage frequently with participants. Lack of widely available public transportation can make it difficult for rural drug court participants to attend the frequent court hearings, treatment sessions, and probation check-ins that drug courts require, putting them at risk for program failure.

"Unfortunately, even if rural counties want to develop alternatives for drug offenders, they often do not have the resources to establish and maintain a drug court independently."

Rural communities too often lack access to adequate treatment services, including residential treatment facilities, intensive outpatient services, medically supervised detoxification, and other evidence-based approaches. Many rural communities lack physicians who are licensed to prescribe and monitor these life-saving medications. While the National Association of Drug Court Professionals (NADCP) provides excellent guidance for vetting providers, rural communities often lack the capacity to monitor unethical prescribers of opioids to treat physical pain. Unfortunately, even if rural counties want to develop alternatives for drug offenders, they often do not have the resources to establish and maintain a drug court independently. Many rural drug courts are significantly underfunded and understaffed.

RURAL DRUG COURT SUCCESS AND STRATEGIES

Innovative strategies are helping rural drug court models become successful. For example, peer recovery advocates can supplement formal treatment. These individuals use their own experience of substance misuse and recovery to support others, help connect drug court participants with supportive services and recovery communities, as well as help transport participants to treatment, court, and other meetings. In Pennsylvania, the Recovery Specialist Program provides one-on-one recovery coaching to individuals in treatment across a five-county area. The program is designed to assist individuals to stay in treatment by matching them with seasoned recovery veterans to work together and build recovery capital.

Another innovative approach used by rural drug courts is teleservices, which is technology that transforms how courts deliver remote treatment and facilitates remote supervision of participants. Rural drug courts throughout the country are experimenting with new technologies that allow participants to participate in treatment remotely, such as videoconferencing, smartphone apps, and portable drug testing devices. Likewise, technology is helping rural drug court participants appear remotely for court hearings and probation check-ins, and even providing participants with drug tests remotely.

“Rural drug courts throughout the country are experimenting with new technologies that allow participants to participate in treatment remotely.”

Lacking consistently available treatment providers, Montana’s Ninth Judicial District Treatment Court, which serves four rural counties and an Indian reservation, has paid treatment providers from a neighboring district to travel there and hold treatment groups. This practice was discontinued due to high costs, and now the court uses videoconferencing technology to provide remote treatment services. The outside treatment providers provide weekly therapy groups, including a Moral Reconation Therapy group. All drug court participants are required to attend these remote sessions. Additionally, the court offers remote anger management sessions and a women’s group.

Similarly, the Klamath County Veterans Treatment Court in Oregon, which serves a population of 68,000 in a land area larger than the State of Connecticut, has scarce access to local treatment services. Court participants use a telehealth link, which is located at a local Veterans Administration community-based outpatient clinic, to access providers across the State and the Pacific Northwest. Available telehealth services include treatment for substance use problems and post-traumatic stress disorder.

ACTION STEPS TO HELP BUILD EFFECTIVE RURAL DRUG COURTS

Building an effective drug court is no longer a process of trial and error. There is an abundance of resources available to provide guidance. The National Drug Court Resource Center provides access to research, publications, and fact sheets that can be utilized for training along with the video lessons housed on the Treatment Courts Online platform. Building an effective drug court in rural communities requires a few essential building blocks to help them succeed. These steps include:

1. **Identify a willing and engaged judge**. An important key to success is the special role of the judge. Serving as a drug court judge introduces a dramatically new role, that of engaging people in treatment, motivating them in treatment, and being willing to reengage them when they slip and fall and fail without blame. This requires a judge who is committed to learning about substance misuse and is prepared for meaningful engagement with drug court participants to steer them toward success. In rural communities, judges preside over the drug court alongside many other functions. One way to convince a sitting judge to become involved is to have him or her visit an existing drug court, observe the process, and interact with the team.

2. **Build a strong dedicated team**. Drug courts employ a collaborative, multidisciplinary team approach. Drug courts are most successful when all key stakeholders—judge, prosecutor, defense counsel, case managers, treatment providers, court coordinators, probation officers, and law enforcement— participate actively and consistently in the program.

3. **Provide ample access to training**. Drug courts require team members to acquire specialized knowledge and skills to work effectively. The team should receive basic training in the psychopharmacology of addiction, substance misuse treatment, behavior modification, and drug court policies and procedures. A few national nonprofit organizations like the National Drug Court Institute, Center for Court Innovation, and Tribal Law & Policy Institute offer no-cost training and technical assistance.

4. **Establish relationships with quality providers**. Drug courts need community-based treatment partners to provide participants with reliable, high-quality treatment and recovery support services, and access to evidence-based treatment including behavioral and psychosocial support. Participants are not positioned for success when they lack necessities, such as housing, food, and transportation, or because they are not allowed to use the best treatment for their specific disorder. Engage the local treatment community and identify all the treatment resources available and build strong partnerships with treatment providers who can provide a range of evidence-based treatment services.

5. **Identify creative ways to fill resource gaps**. When gaps exist in the continuum of services, find ways to pursue creative strategies and work with community partners to identify potential resources. If only one professional is available to serve a large geographic area, one solution could be "circuit-riding" where the professional travels to each community on a regular basis. A contracted treatment provider (treatment counselor) from a larger community could drive to a rural location and hold group sessions both before and after the drug court docket. Coalitions, places of worship, and civic groups could provide resources as well. Counseling space (office space) could be available through a local faith-based organization. Also, consider sharing resources between rural courts, utilizing peer recovery advocates, and teleservices to help overcome resource limitations.

6. **Encourage community engagement and community buy-in**. Find ways to integrate the community to help them to see the benefit. For example, hold a community partners meeting and include individuals from law enforcement, community groups, hospitals, churches, and mental health services. Make community service an important aspect of the program or have drug court participants volunteer at community events to earn community service hours. Efforts to enlist and maintain community support through public presentations by the judge and other team members, as well as media coverage of graduations and other positive events are important.

TN ROCS DOCKET: AN ALTERNATIVE STRATEGY TO MANAGE LOW-RISK, HIGH NEED DRUG OFFENDERS.

Tennessee's Recovery Oriented Compliance Strategy (TN ROCS) is an intervention strategy for working with drug offenders who have an urgent need for treatment but may not qualify for drug court programs. The program's founder Judge Duane Slone based the unique strategy, which helps reduce State jail populations and confronts the opioid-driven addiction crisis, on three components—adequate assessment of treatment, frequent accountability, and "a big stick." Paths to TN ROCS include: those not accepted or ineligible for Drug Recovery Courts (DRC), Pre-trial release, Probation Violations, either by committing new crimes or "technical" violations, or as part of an Original Sentence. Placement on the TN ROCs docket is at a judge's discretion; it may be voluntary or involuntary. TN ROCs have fewer steps, allowing individuals to more quickly be integrated into treatment. In contrast, drug recovery court programs are resource-intensive, often requiring many volunteers to operate, and participants must complete a highly-structured, four-phased recovery process. TN ROCS draws inspiration from this model but offers a less resource intensive approach. For example, TN ROCs does not require the same extensive staff or drug court team involvement, and only requires a judge to begin handling this portfolio of cases within their docket. Still, the program requires buy-in and hard work from participants who want to succeed.

Drug Recovery Courts vs. TN ROCS

	Drug Recovery Courts	TN ROCS
**Treats High Risks for Recidivism**	X	X
**Treats High Needs for Behavioral Health & Other Services**	X	
**Application Required (Voluntary Participation)**	X	
**Utilize Three Essential Components of DRCs**	X	X
**No Eligibility Restrictions for Sentences Permitting Probation**		X
**Highly Dependent on Justice System Volunteers**	X	

TN ROCS participants are usually non-violent offenders with demonstrated, persistent substance abuse problems who cannot seem to break the cycle of their own addiction. The participants are partnered with criminal Justice liaisons who perform health assessments, draw up evidence-based behavioral health treatment plans, which often consist of intensive outpatient therapy and medication-assisted treatment; and arrange for the acceptance of the offender with all treatment and recovery support providers. The behavioral health treatment plan continues to be a condition of probation and therefore the sentence. Once added to the TN ROCS docket, members meet regularly with corrections officers, who monitor their adherence to the behavioral health treatment plan. Docket members also make regular appearances before a judge to provide progress updates. The more progress completed and the longer participants adhere to the requirements of their treatment plans, the less frequent these appearances. Eventually, participants can complete the program with approval from a judge. The length of time on the Docket is open ended, however, it typically takes two years before the person no longer has to report to the judge.

Three Necessary Requirements For TN ROCS

CRIMINAL JUSTICE LIAISON

Trusted Person or Organization Provides Accurate Screening, Assessments, and Referrals to Appropriate or Best Available Level of Treatment.

COMMUNITY CORRECTIONS OR CASEWORKER

Trusted Person or Organization Provides Appropriate Level of Supervision.

JUDICIARY

First, Affirmation by the Judge is Critical to the Success of the Individual. Second, the Individual Appears Before the Court to Review Compliance with the Behavioral Health Treatment Plan and to Make Adjustments to Terms and Conditions of Supervision Where Necessary.

One of the docket's biggest successes is the high rate of healthy babies born to young mothers participating in the TN ROCS program. All 34 babies born during the first three years of the program were healthy, and 30 of the mothers were able to retain custody of their children. That rate far exceeds the average for women with behavioral health issues who are involved in the criminal justice system.

Section 5

Recovery

PARTNER INSIGHT AND RECOMMENDED ACTION STEPS

Once people get treatment, there are many services needed to ensure they stay healthy in long-term recovery. From housing to transportation and employment training, there is a significant gap in recovery supports in rural communities. Yet, there are also strong assets in rural America for local leaders to tap in building these supports. For example, in most small towns, the faith community is a prominent piece of the local support network that comes together around any other challenge the community may face.

Recovery is a lifelong process that is most successful when carried out in community. To build a recovery-friendly community, rural leaders must be proactive and take action to learn about the needs of a person in recovery, take inventory of community assets and gaps, and foster a network of stakeholders to create an environment in which people in recovery can live substance-free.

HOW FAITH COMMUNITIES CAN HELP RURAL COMMUNITIES ADDRESS SUBSTANCE USE DISORDER

By USDA, Center for Faith-Based and Neighborhood Partnerships

ROLE OF FAITH-BASED COMMUNITIES

The American people are key drivers of fundamental change in our country, and few institutions are closer to the people than faith-based and community-based organizations. Faith leaders play an integral role in bringing communities together, advancing a culture of compassion, and partnering meaningfully to leverage the vast array of resources and deliver critical services in our communities.

Faith communities play a vital role in education, prevention, and treatment related to the opioid crisis at a very local level. These groups have been a caring and driving force in assisting local communities to weather many storms in our country's history, including helping to heal people from the negative effects of war, pandemics, natural disaster, economic downturn, and social upheaval.

Since 2002, the USDA Center for Faith-Based and Neighborhood Partnerships has recognized the need and supported efforts to engage a broad and inclusive array of faith-based community-based organizations to deliver vital services in our communities. Currently, the center works to empower faith communities and leaders to navigate USDA programs and resources to support those who are struggling with SUD, including opioids misuse and its dangerous consequences.

FAITH COMMUNITIES ARE LEADING LOCAL EFFORTS TO TACKLE SUBSTANCE USE PROBLEMS

Today many faith leaders are leading the charge to serve their communities in response to the opioid crisis. These leaders have highlighted the many ways that faith communities are opening their doors to those in need during the height of the opioid crisis. Their work shows how faith-centered recovery models can help combat addiction.

Faith communities provide many types of support for opioids use disorder. Informal methods can range from rallying around community members or offering a moral uplift, to assisting in fulfilling unmet needs. Formal services can include counseling, transportation support, and serving as a drop-off site for unused medications. They can encourage congregations to focus on educating and reaching their members to connect them to recovery and addiction programs.

As the opioid crisis persists, houses of worship can increase their commitment to reduce the devastating effects of this crisis on its victims and their families. This includes supporting a judgment-free and compassion-centered approach, promoting and supporting healing through direct care, forgiveness, hospitality, and friendship, and actively participating in community-wide strategies.

INCLUSION OF FAITH-BASED LEADERSHIP

Faith communities constitute a prominent segment of society and often have a front-row view of the opioid crisis and its devastating impacts. They bring both theological and social resources to respond to these challenges. By their very nature, faith-based organizations are embedded in neighborhoods and serve as trusted pillars among community members. Additionally, many of these interfaith organizations provide a wide variety of social service programs to help their communities.

Accordingly, faith leaders should be included and supported as a member of any local task force or working group formed to address the crisis right alongside political leadership, law enforcement, healthcare providers, as well as educational institutions. Active inclusion and participation will strategically leverage best practices, models, existing resources, and programs to empower and assist communities in addressing opioids misuse.

Given the assets and untapped potential of faith communities, it is essential that their leadership be included along with secular service providers, public and private funding agencies, support systems, and consumers of behavioral health services in crafting an effective behavioral health service delivery system.

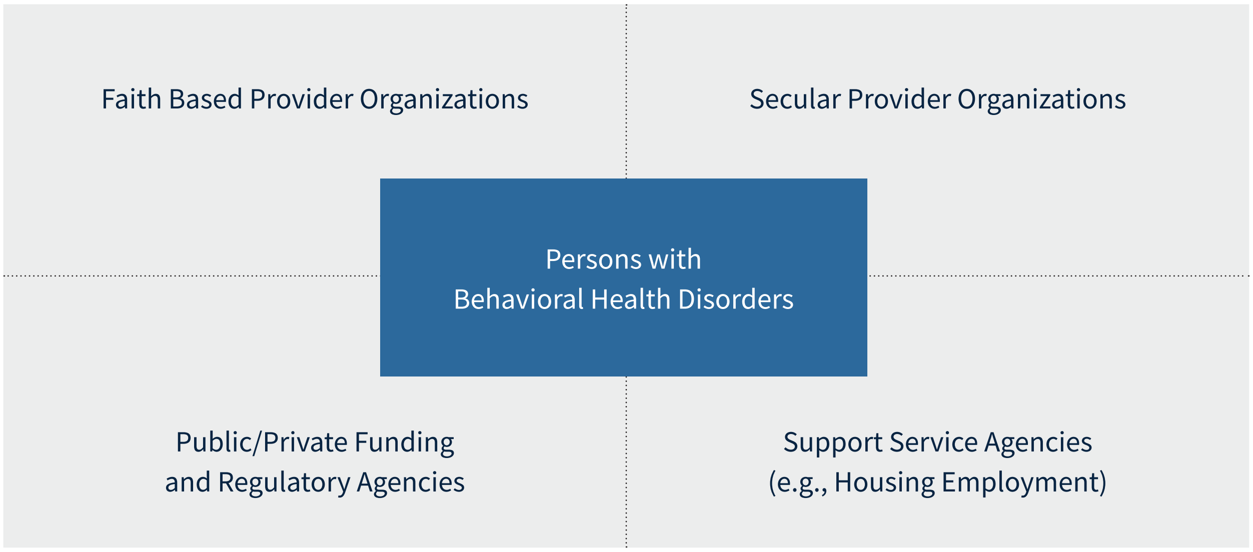

Faith leaders' institutional role as trusted community leaders is essential to building bridges within and in between communities. They function as critical information conduits, which are necessary for communicating constituent needs to local, State, and national leaders and ensuring that the community receives relevant and much-needed information and resources.

While the challenges appear to be many, there are a variety of practical strategies faith communities can undertake to serve their respective communities. These activities seek to address concerns primarily through the growth and development of social capital and social networks in the community. By working with others to identify needs and gaps, faith communities can also leverage their other assets to assist families with transportation, housing, family and youth mentoring, and job coaching. These support networks will enable faith communities, in partnership with local authorities and partners, to assist both prevention and response efforts.

ACTION STEPS FOR FAITH-BASED COMMUNITIES TO ADDRESS SUBSTANCE USE PROBLEMS

To break the cycle of addiction, faith communities should work together with community partners, families, and one another. Together, this effort offers an opportunity to make a lasting change and save lives. These practical activities can assist faith-based communities in addressing the opioid crisis.

1. **Form an interfaith group to avoid working in silos.** Appoint representatives to interact with the community taskforce or working group to gain an understanding of the community vision for change and how the interfaith community can help maximize resources and increase the impact.

2. **Educate the interfaith community about the breadth and scope of the challenge and the local level of substance use and behavioral health issues**. Host meetings with interfaith partners and take steps to better understand what is most and least helpful for bringing healing to those who are affected by opioid use.

3. **Partner with local health organizations and service providers to stay up to date on the latest information, statistics, and educational resources**. Maintaining a close relationship with local health providers will ensure that the community is up to date on valuable trends and treatment options and can assist with the education of lay members and congregants.

4. **Assess the interfaith community assets**. Determine which partners have a high number of available volunteers, consistent access to vehicles to supplement transportation need, or available meeting space for counseling.

5. **Work with partners to educate and train lay people and pastors to deal with addiction**. Providing congregants and faith leaders with formal training in convening and partnering, facilitating, breaking down the barriers of stigma, and addressing substance misuse in communities, will create enabling environments for successful interventions. Educating the community around critical issues or topics in behavioral health will build consensus and identify the most suitable local response.

BUILDING A STRONG RECOVERY COMMUNITY IN RURAL AREAS

By National Alliance for Recovery Residences

Recovery is a person-driven process with many pathways to health. The process is more successful when implemented within a supportive structure, such as a recovery community. These are networks of individuals who share the common goal of lifelong sobriety and offer support and fellowship to their peers. Due to high rates of SUD, many types of recovery communities have sprung up across the country to support persons in recovery. These range from sober living facilities and recovery residences to twelve-step support groups such as Alcoholics Anonymous (AA), Heroin Anonymous (HA), Cocaine Anonymous (CA), and Narcotics Anonymous (NA). Recovery community organizations (RCO) are independent, nonprofit organizations led and governed by local representatives. They focus on the needs of individuals, families, and communities seeking or in recovery from SUD.

FACTORS THAT DRIVE RECOVERY CARE

Recovery services include the provision of continuing care following treatment, education regarding self-care, regular check-ups, and linkage to community resources and support resources such as recovery mutual aid meetings and recovery homes. SUDs typically require long-term involvement with the healthcare system. In rural communities there are fewer health practitioners and both mental health and SUDs services are harder to obtain. The barriers to recovery in rural areas typically include:

- Limited funding for recovery residences
- Transportation challenges
- Limited access to a continuum of care/recovery-oriented systems of care
- Lack of detoxification facilities
- Lack of mental, dental, and health services
- Limited access to transition and long-term housing
- Lack of anonymity
- Limited access to mutual-aid meetings
- Limited access to employment

Four factors impact rural behavioral healthcare and drive the delivery of SUD recovery support services to the community. These variables determine when and if rural residents with behavioral healthcare needs will seek care.

- **Availability** includes basic and specialized recovery support services and staffing that can deliver services.
- **Accessibility** is when and where services can be received and the coordination of services across the behavioral health and social service system. This also includes transportation issues that may be involved.
- **Affordability** includes the costs of receiving care and availability of benefits, insurance, and alternative revenue streams to underwrite services.
- **Acceptability** addresses the consistent issues around stigma for those who need services. In addition, rural residents may be more likely to make use of informal supports, such as neighbors, family, churches, and other community groups.

FOCUS ON BUILDING RECOVERY ORIENTED SYSTEMS OF CARE IN RURAL COMMUNITIES

Traditional service systems are not sufficient to handle recovery-oriented services. Instead it is necessary to change the service system structure to bring about a truly recovery-oriented service system. A ROSC (Recovery Oriented Systems of Care) is a coordinated network of community-based services that support person-centered, self-directed approaches by building on the strength and resilience of individuals, families, and communities to take responsibility for their own sustained health, wellness, and recovery. The central focus of a ROSC is to create an infrastructure or, system of care, with the resources to effectively address the full range of substance use problems within communities. A ROSC should provide a network of services and supports that addresses the totality of substance use problems.

Examples of Recovery-Oriented Activities

Prevention	Intervention	Treatment	Post-Treatment
• Early screening before onset<br>• Collaborate with other systems, e.g., child welfare, VA<br>• Stigma reduction activities<br>• Refer to intervention treatment services	• Screening<br>• Early intervention<br>• Pre-treatment<br>• Recovery support services<br>• Outreach services	• Menu of treatment services<br>• Recovery support services<br>• Alternative services and therapies<br>• Prevention for families and siblings of individuals in treatment	• Continuing care<br>• Recovery support services<br>• Check-ups<br>• Self-monitoring

Bringing the ROSC approach to rural communities means examining these areas through a recovery-focused approach to make local addiction recovery support services that are available, accessible, affordable, and acceptable. The hub and spoke model consists of a network of recovery support providers in metro areas who deliver local services in rural areas through the support of a larger hub provider, who has committed resources in a variety of forms in the rural market to address treatment and recovery support services quality.

Rural leaders can convene a community task force and develop a plan to address the barriers to a thriving recovery community. Rural communities can also reach out to nonprofit organizations that specialize in addiction recovery, collaborate with local faith-based and civic groups, and apply for Federal grants to help bridge the gap. Another solution is to introduce new and innovative delivery models and adapt broader e-connectivity to increase access to web-based health technology resources, such as telemedicine, telehealth, health apps on smart phones and tablets, online recovery coaches, and other programs. Relationships between health professional schools and Critical Access Hospitals can also create rural behavioral health practice training sites.

INCREASE ACCESS TO COORDINATED CARE IN RECOVERY HOUSING

Recovery homes can be a viable and cost-effective alternative to established recovery-oriented systems of care. Often when rural communities offer affordable housing options and addiction treatment services, these services are rarely coordinated. This lack of coordination leads many people in early recovery to return to environments that foster addictive lifestyles, increasing the likelihood of relapse or continued substance use. Recovery homes are a good alternative to ROSC because they provide safe and healthy environments that support residents in their recovery. Increasing the availability of recovery housing ensures that the system of care is responsive to various needs at different points in the recovery process.

Recovery homes offer mutual help-oriented, financially self-sustaining, communal living environments where individuals in recovery can live after inpatient treatment or incarceration, during outpatient treatment or as an alternative to treatment. When people are newly sober, they are moving from a culture of addiction to a culture of recovery. Recovery housing gives them the time and support needed to change those behaviors that were required for life and death survival in the culture of addiction. Individuals build resources while living in a recovery residence that will continue to support their recovery as they transition to living independently and productively in the community. There is a greater concentration of recovery homes in urban and suburban areas. Where they exist, rural residences tend to provide higher or a more comprehensive mix of support because there are fewer support resources in the surrounding area. They also tend to be more closely linked with a faith-based community, treatment center, or other supplemental support community.

There are four levels of recovery residences as certified by the National Alliance for Recovery Residences (NARR). The services range from peer-to-peer recovery support (all recovery residences) to medical and counseling services (recovery residences offering higher levels of support). One level of support is not considered better than another. They simply differ in the kind and intensity of services and supports provided. For example, at a minimum, a Level 2 recovery residence provides a sober, safe living environment coupled with peer recovery support through a social model recovery philosophy and structure. In contrast to a Level 1 residence, a Level 2 residence has an owner/operator appointed house manager.

Recovery Residence Levels

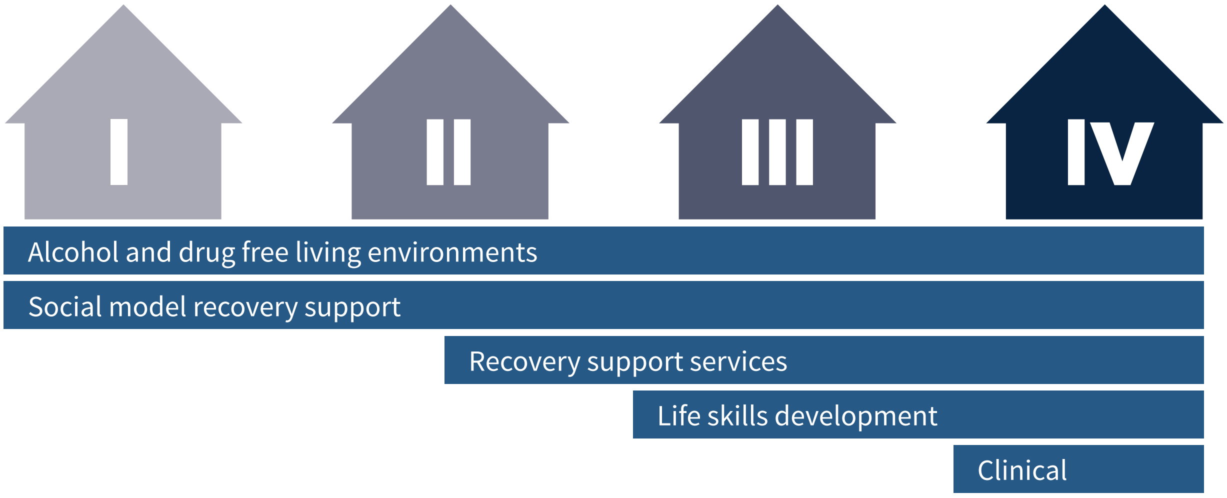

ACTION STEPS TO FOSTER STRONG RECOVERY SYSTEMS

Maintaining a safe, stable recovery community in the earliest stages of sobriety as a foundation for lifelong recovery in rural places with limited resources is key. In the early stages, it is critical to connect people with SUD to mutual-aid groups, either faith-based or 12-step fellowships. A few ways to foster stronger recovery systems or recovery residences in rural communities include:

1. **Survey available recovery housing capacity**. Increasing the availability of recovery housing ensures that the system of care is responsive to various needs at different points in the recovery process.
2. **Seek out creative resources for funding**. High start-up, operating, and capital expenses can be a significant barrier to creating and sustaining quality recovery residences. Revenue streams will vary by type of residence; there may be programs for certain targeted populations such as criminal justice clients or voucher programs and insurance benefit packages that can be tapped.
3. **Identify supporting roles for community members**. Everyone can play a part in the fight against SUDs. In a rural community there is a higher reliance on family, extended family, social networks, and local institutions to solve problems.
4. **Integrate recovery housing more fully into healthcare and other systems**. Consider delivering addiction treatment services through rural satellites, co-locating with existing health and social service resources and assertive outreach programs.
5. **Engage in regional collaborations**. Planning on a regional basis allows geographically contiguous rural areas to create and sustain recovery supports jointly.
6. **Consider innovative treatment delivery models**. Individuals need various types and levels of support at different stages of recovery. Matching an individual with the appropriate level of support is both recovery supportive and cost effective.
7. **Support peer-based recovery support services**. During recovery coaching a peer mentors an individual seeking recovery by helping set goals, develop a plan, and serve as a role model during recovery. This style of coaching includes helping connect the individual to recovery support resources needed to restructure life, such as professional and non-professional services including housing and employment. The coach also serves as an advocate and liaison to formal and informal community supports, resources, and recovery supporting activities.

MOBILIZING THE RECOVERY COMMUNITY IN RURAL AREAS

By Faces & Voices of Recovery

THE ADVANTAGES OF PEER SUPPORT SERVICES

Peer Recovery Support is a social support service designed to fill the needs of people in or seeking recovery. People who have similar experiences with SUDs give others encouragement, hope, assistance, guidance, and understanding, all which aids in lifelong recovery. Peer recovery support specialists, often called recovery coaches, serve as a bridge between individuals and resources in the community. They offer wide-ranging nonclinical services that assist individuals and families working toward recovery from substance use conditions. These include social supports and services such as employment services, housing, peer coaching, and substance-free social and recreational activities.

Despite bringing high value, recovery support services are often insufficiently funded throughout the prevention, treatment, and recovery continuum of care, largely due to funding constraints and a lack of information, capacity, and infrastructure. Yet studies on peer recovery supports have demonstrated reduced relapse rates, increased treatment retention, improved relationships with treatment providers and social supports, increased satisfaction with the overall treatment experience and increases in quality of life factors.

Key Advantages of Peer Support Services

- Reduces relapse
- Reduces homelessness
- Engages people into care
- Helps individuals and families initiate and stabilize early recovery and sustain long-term recovery
- Improves continuity of care
- Provides meaningful cost savings
- Reduces the use of acute care services such as emergency rooms and detoxification centers
- Helps people move beyond treatment settings and find support in the communities where they live, work, and play

PEER SUPPORT SERVICES IN RURAL COMMUNITIES

Not surprisingly, providing recovery supports in rural communities is challenged by physical, emotional, and social isolation, as well as multiple vulnerabilities that coexist with addiction (e.g., trauma, health, problems, homelessness) coupled with fewer opportunities for individual self-sufficiency. Two common barriers that place rural residents at a distinct disadvantage for initiating or maintaining SUD recovery are the limited availability of services and the cost of care. Yet rural communities have their own rich internal assets that can be mobilized to expand recovery supports and a strong history of resilience.

RCOs are community-based nonprofits that typically facilitate the placement of peer recovery support workers in outreach positions. RCOs advocate for the meaningful representation and voice for people in recovery and their families at local, State, and Federal policy levels on issues that affect their lives. Strategic placement of peer-based community support embedded within frontline services offers a timely opportunity for intervention.

Many rural communities are starting to organize strong grassroots RCOs and linking them into a national movement led by the Association of Recovery Community Organizations (ARCO) at Faces & Voices of Recovery. These RCOs are developing recovery leaders and offering opportunities for the recovery community—people in recovery, family members, friends, and allies—to express their collective voice on issues of common concern, respond to community-identified recovery support needs, and provide a forum for recovery-focused community service. However, the expansion of RCOs into many rural communities is still limited, mainly because of insufficient financial resources.

In recent years, the number of RCOs has risen dramatically to meet the high demand for peer-based recovery support services. An RCO is an independent, nonprofit organization led and governed by the recovery community, providing advocacy, education, and/or peer-based recovery support services. These non-clinical services, designed and delivered by the recovery community, include peer recovery support specialists, telephone-based recovery support, mutual aid groups, recovery housing, and much more. When an RCO operates a recovery community center, that center becomes the hub of recovery activity where individuals can volunteer, seek employment and education, and participate in substance-free recreation activities.

THE RURAL PEER SUPPORT MODEL

Key Features of a Rural Peer Recovery Support Model

- Connects individuals with SUD to community-based care
- Strong Recovery Community Organization that provides advocacy, education, and peer recovery support services
- Boots-on-the-ground peer recovery team
- Capitalizes on community bonds and indigenous institutions
- Able to break down the stigma that prevents people with SUD and OUD from seeking services
- Forges community partnerships
- Cultivates volunteerism

The optimal model for rural peer recovery support is a strong, vibrant recovery community organization providing advocacy, education, and peer recovery support services. This should capitalize on rural community ties by focusing on indigenous recovery resource development closely aligned with the rural reliance on family, extended family, social networks, and indigenous institutions (e.g., churches, co-ops) to solve problems rather than relying on service professionals. Many people seeking recovery in rural communities find support from family, friends, primary care physicians, clergy, faith communities, work, school, and through local and online recovery support groups. There is also great potential for cultivating volunteerism within rural communities of recovery. The peer workforce brings their own relevant experience to share with those in need, and they are breaking down the stigma that keeps many from reaching out for help.

When the recovery community is not at the table, policies and programs risk becoming ineffective and inadequately designed or delivered. Rural services may be inaccessible or unattractive to the needs and interests of the people they intend to serve. As such, the most critical need in rural communities is education of key community leaders and the community at large about recovery and peer-based recovery support services. Once buy-in is achieved, mobilization of the local recovery community can be rapid, particularly if the people leading the local effort are well-known and trusted community members. In rural communities, the speed of this potential mobilization is enhanced because everyone

knows each other, rural agencies work well together, and people are likely to wear multiple hats. Trust is essential and, when that trust is present, most rural communities are willing to step forward and offer their full support of new recovery support initiatives.

Although research is limited on the ideal design, delivery, and evaluation of recovery support services specifically for rural communities, several initial examples show promise as model rural programs. Successful rural RCOs are taking a lead role in educating the public, policymakers, and service providers about the prevalence, pathways, and styles of long-term addiction recovery. They are engaging in discussions about the multiple pathways of recovery and sharing their stories publicly to eliminate the stigma and discrimination often faced by people in recovery.

- **The Green Mountain State.** The Vermont Recovery Center Network currently operates nine independent member centers and three affiliate centers. The Recovery Community Centers have been established for the provision of recovery support services in communities throughout Vermont. Recovery centers are local, consumer driven, non-residential facilities that provide peer supports, sober recreation activities, volunteer opportunities, community education, and recovery supporting services. Leadership is provided by an executive council that consists of a board member from each recovery center. The council has been tasked by Vermont's legislature with advising the Vermont Department of Health in prioritizing services, considering funding needs for recovery centers, reviewing recovery center funding proposals, and providing recommendations for disbursement of funds for the support of recovery centers.
- **Making Taos a Recovery-Friendly Community**. Taos County, New Mexico is a beautiful place to live and a wonderful community. The recovery community has come together because they care about making Taos a community where recovery is something to be proud of, not ashamed of, where there is help available for people and families struggling with SUDs, and where no one is ashamed to ask for help. Recovery-Friendly Taos' mission is dedicated to promoting a recovery-friendly community through development of supports, opportunities, and access to services, education, and information. They are committed to reducing the barriers created by stigma and replacing it with hope and well-being.
- **Idaho Recovery Community Centers.** The Idaho Association of Recovery Community Centers (IARCC) is a consortium of nine recovery centers providing a broad spectrum of recovery support services across the State's 44 counties (37 of which are rural or frontier). Each center operates with one or two staff members and a larger volunteer workforce (more than 23,000 volunteer hours were logged across the centers this past year). Funding for the centers comes from State grants and private local donations. The centers provide service coordination with local health and human service agencies, jail outreach, recovery support groups, recovery-focused classes, and workshops for individuals and families, recovery coaching, telephone-based recovery checkups, and sober social events. These centers averaged more than 5,000 visits each in the fiscal year 2016-2017.
- **Western Massachusetts**. The RECOVER Project in Greenfield is a safe, welcoming community that supports recovery by sharing the wisdom of lived experience and strengthens the community through full participation. They create resource connections; advocate to overcome barriers and promote the reality that recovery is possible for all. The RECOVER Project is committed to serving as a resource for information about addiction and recovery.

ACTION STEPS TO MOBILIZE THE PEER SUPPORT COMMUNITY

The National Recovery Institute provides technical assistance and training for local and State entities on the development of peer recovery support services by drawing on the expertise of RCO leaders across the nation. National standards for the delivery of peer support services have been established by the Council on Accreditation of Peer Recovery Support Services. Here are some concrete steps that local leaders in rural communities can take to mobilize the peer support community.

1. **Bring peer support specialists to the table early to create a shared vision of recovery**. To ensure that the voices of those with the experience of addiction recovery are heard and respected, they should be involved at all levels of decision-making, program, policy, and strategic planning.

2. **Identify leaders in the recovery community**. Use community organizer roles (versus clinical roles) to develop and mobilize indigenous recovery support resources within rural areas.

3. **Identify recovery champions to support the effort and be ambassadors for the cause**. These can include local leaders in the county government, faith community, health department, business leaders, law enforcement, and others.

4. **Launch community visioning**. Hold stakeholder meetings across the state or region to find out what the recovery community wants, what their hopes of the future are, who will be involved, and how the RCO will be structured.

5. **Assess community needs; identify the needs, gaps, and strengths in your community**. This will help decide which types of supports, activities, or service to provide.

6. **Conduct a needs assessment to identify areas where SUD and related harms are most prevalent**. The number and location of opioid treatment providers in the areas, including providers that offer OUD services; all existing activities and their funding sources in the State that address opioid use prevention, treatment, and recovery activities and remaining gaps in these activities.

7. **Get creative**. Capitalize on the indigenous recovery capital that exists or that can be mobilized within rural areas.

8. **Create recovery community centers that make recovery visible on Main Street**. These should provide a setting for the delivery of non-clinical, peer-based recovery support services, supports, and activities.

9. **Celebrate recovery from addiction**. Plan public recovery celebration events (e.g., marches, rallies, concerts) that offer living proof of the transformative power of recovery.

INCREASING HOUSING OPTIONS FOR PERSONS IN RECOVERY IN RURAL COMMUNITIES

By Housing Assistance Council

SAFE AND STABLE HOUSING

When it strikes, SUD and opioid misuse often negatively impacts every part of a community. Responding solutions must then positively address each of those impacted sectors for the region to recover and thrive. This includes not just essential medical treatment and greater access to quality healthcare, but also long-term elements of recovery such as providing stable, safe, affordable housing options.

The home should be a calm and safe space with a stable, substance-free environment. Unfortunately, in rural areas there is a shortage of affordable housing that meet these basic requirements. Those who complete treatment for SUD often return to communities and housing situations where they first became addicted, creating an unhealty cycle. Safe and stable housing is essential for any addiction treatment and recovery services to be effective.

RURAL HOUSING BARRIERS

Common housing circumstances related to SUDs often overlap with housing challenges for rural homelessness: reentry from the criminal justice system, household stress, and access to housing services. People recovering from SUDs may need assistance in acquiring the life skills required to become law-abiding, sober citizens. Substance use rehabilitation, vocational training, and employment programs can all help achieve these goals.

"Those who complete treatment for SUD often return to communities and housing situations where they first became addicted, creating an unhealthy cycle."

The limited supply of quality rental and supportive housing is a consistent, overriding housing challenge in many rural communities, and around the country. Housing access and affordability is especially problematic for low-income households and renters in rural areas nationwide. Housing costs tend to be lower in rural areas, but wages are also lower, resulting in an increasing number of rural households who struggle to pay monthly housing expenses. These affordability constraints are worse for people recovering from addiction and dealing with challenging economic circumstances.

Homelessness often results from a combination of substance misuse and mental health issues, which creates a complex community challenge that demands a tailored approach. Housing and homelessness manifest differently in rural environments. Highly effective urban solutions aimed at homelessness too

often falter in rural America. Here nonprofits, not governments, often provide services and those who are homeless are often less conspicuous but still need assistance. Rural homelessness recognizes no geographic boundaries, and counties with entrenched poverty deal with it regularly. Limited availability of rental housing, discrimination against those battling opioid addiction, and lower rates of adequate health insurance compound the problem.

Persons in recovery must have the ability to live near a treatment facility or a recovery center to receive consistent services for addiction. Since recovery centers or treatment facilities may not be open in every area of a rural region, affordable housing absolutely must be available where they do exist. Housing is an essential bedrock for day-to-day life in recovery. Once this critical piece is settled, there is more time and emotional energy for recovery and treatment efforts. After recovery, a stable environment without negative influences can help ensure successful recovery and prevent relapses.

WHERE HOUSING FITS INTO THE SOLUTION

During a recent congressional hearing entitled “The Role of Federal Housing and Community Development Programs to Support Opioid and Substance Use Disorder Treatment and Recovery” on August 16, 2018, experts testified why housing programs are an important solution to the opioid crisis. The testimony included this statement: “Stable housing is a basic human need and one of the primary social indicators of public health. Access to stable housing is one common factor that is essential for all these strategies to produce positive results.”

“Stable housing is a basic human need and one of the primary social indicators of public health. Access to stable housing is one common factor that is essential for all these strategies to produce positive results.”

Tackling the opioid crisis is a multifaceted problem that requires a multifaceted approach. Recovery from opioid addiction requires treatment, effective treatment requires safe and stable housing, safe and stable housing requires employment, and employment requires effective treatment and staying in recovery. If even one element fails, progress will slow to a halt.

Resources may come from many directions, but their success will depend on local communities and local resources. Educating teachers, parents, healthcare providers, and local government officials is an important step towards solving the opioid crisis. Expansion of treatment and addiction support groups will not be effective without strong community understanding that addiction is a physical dependency, not a moral failing. Addiction must be viewed as a disease, rather than a moral shortcoming. Local opinion can help create a sense of acceptance, reduce stigma, and increase the opportunity for recovery.

ACTION STEPS TO PROVIDE COMMUNITIES WITH STABLE HOUSING

1. **Get the data**. Take steps to find out how affordable housing intersects with the opioid crisis in your community and the areas with the greatest need.

2. **Bring the housing community to the table.** Bring housing representatives from the State or local offices and non-for-profits to the table early. People need to be in a stable, drug-free environment to complete the recovery process, but there is often a shortage of safe and drug-free affordable housing in rural communities. Invite housing stakeholders to join working groups or the taskforce that is formed to address the opioid crisis in the community to get a jump-start on tackling the housing issues.

3. **Go local**. Reach out to your local or community-based housing nonprofit organization for advice. Local housing nonprofits understand the unique housing needs of the community and the best resources available. Creating a stable and consistent home can often lay the groundwork for recovery in the battle against addiction. Reaching out to local housing nonprofits can be the first step towards finding this stability.

4. **Create a local resource guide**. Many jurisdictions compile local resource guides for those in the treatment and recovery phases of substance misuse. For example, the Virginia Department of Health developed a resource guide in response to the opioid emergency, which provides links and contact information for shelters, support groups, inpatient and outpatient facilities, websites, helplines, and hotlines. Local guides like these can simplify the confusing process of identifying resources and increase the chance that those in need quickly find the necessary resources.

5. **Collaborate with community leaders**. The opioid epidemic is a multi-faceted problem that requires multifaceted cross-agency coordinated solutions. Help local social service providers, criminal justice officials, the medical community, and housing providers connect and work together. Cross collaboration across key sectors is integral. Serve as the catalyst to help connect these sectors in the community.

6. **Advocate for rational solutions**. Stigmatization and excessive judicial responses to addiction can exacerbate the effects of the opioid crisis. Background checks are necessary and important, but blanket restrictions for criminal convictions often prove counterproductive in this new environment. Individual assessment of tenant requirements in both private market and federally funded housing can increase access to quality and positive housing arrangements for those recovering from substance misuse.

Closing Thoughts

The insight and recommended action steps in this guide are intended to create a roadmap for action in rural America. To find illustrations of actions taken, there are numerous examples of promising practices on the USDA webpage which are meant to help rural leaders visualize what meaningful action in their community could look like. With this information, local leaders are better equipped to shape the future of their community.

The magnitude of the addiction crisis and its impact on rural America will take leaders at the Federal, State and local level working together and each doing their part to build strong, healthy, and drug-free communities. There is no time to waste. The future of rural America is at stake.

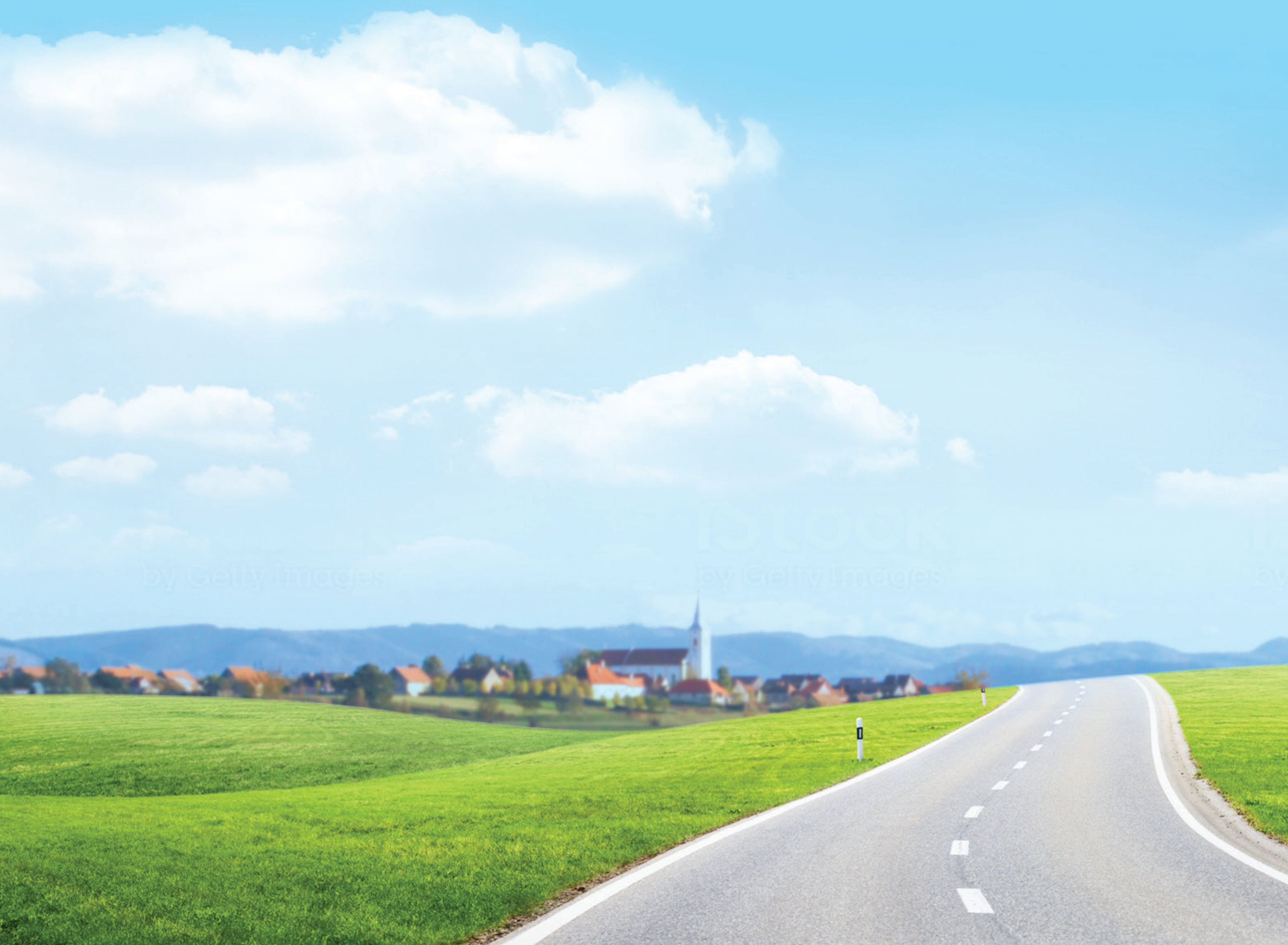

Rural Community Action Guide Partners

Addiction Policy Forum

Addiction Policy Forum is a nationwide nonprofit organization established in 2015 as a diverse partnership of organizations, policymakers, and stakeholders committed to working together to elevate awareness around addiction, and to improve programs and policy through a comprehensive response that includes prevention, treatment, recovery, and criminal justice reform. **https://www.addictionpolicy.org/**

American Farm Bureau Federation

Farm Bureau is an independent, non-governmental, voluntary organization governed by and representing farm and ranch families united for the purpose of analyzing their problems and formulating action to achieve educational improvement, economic opportunity, and social advancement and, thereby, to promote the national well-being. Farm Bureau is local, county, State, national, and international in its scope and influence and is non-partisan, non-sectarian, and non-secret in character. Farm Bureau is the voice of agricultural producers at all levels. **https://www.fb.org/**

Appalachian Regional Commission

The Appalachian Regional Commission (ARC) is a regional economic development agency that represents a partnership of Federal, State, and local government. Established by an act of Congress in 1965, ARC is composed of the governors of the 13 Appalachian States and a Federal co-chair, who is appointed by the president. Local participation is provided through multi-county local development districts. **https://www.arc.gov/**

Center for Court Innovation

The Center for Court Innovation seeks to help create a more effective and humane justice system. Originally founded as a public/private partnership between the New York State Unified Court System and the Fund for the City of New York, the Center for Court Innovation conceives, plans, and operates programs that seek to test new ideas, solve difficult problems, and achieve system change. Center projects include community-based violence prevention projects, alternatives to incarceration, reentry initiatives, and court-based programs that reduce the use of unnecessary incarceration and promote positive individual and family change. **https://www.courtinnovation.org/**

Community Anti-Drug Coalitions of America

Community Anti-Drug Coalitions of America (CADCA) is a nonprofit organization that is committed to creating safe, healthy, and drug-free communities globally. Since 1992, CADCA has demonstrated that when all sectors of a community come together, social change happens. CADCA represents over 5,000 community coalitions that involve individuals from key sectors including schools, law enforcement, youth, parents, healthcare, media, and others. We have members in every U.S. State and territory and more than 30 countries around the world. The CADCA coalition model emphasizes the power of community coalitions to prevent substance misuse through collaborative community efforts. CADCA believes that prevention of substance use and misuse before it starts is the most effective and cost-efficient way to reduce substance use and its associated costs. **https://www.cadca.org/**

Faces & Voices of Recovery

Faces & Voices of Recovery is dedicated to organizing and mobilizing the over 23 million Americans in recovery from addiction to alcohol and other drugs. They promote the resources to recover through advocacy, education, and by demonstrating the power and proof of long-term recovery. **https://facesandvoicesofrecovery.org/**

Housing Assistance Council

The Housing Assistance Council (HAC) is a nonprofit corporation located in Washington, DC with regional offices in the Southeast, Midwest, and Southwest. HAC has been helping local organizations build affordable homes in rural America since 1971. HAC assists in the development of both single- and multi-family homes and promotes homeownership for working low-income rural families through a self-help, “sweat equity” construction method by emphasizing local solutions, empowerment of people in poverty, reduced dependence, and self-help strategies. HAC offers services to public, nonprofit, and private organizations throughout the rural United States and maintains a special focus on high-need groups and regions, such as Indian country, the Mississippi Delta, farmworkers, the Southwest border, and Appalachia. **http://www.ruralhome.org/**

National Alliance for Recovery Residences

The National Alliance for Recovery Residences (NARR) is a 501-c3 nonprofit organization dedicated to expanding the availability of well-operated, ethical and supportive recovery housing. NARR developed the most widely referenced national standard for the operation of recovery residences. NARR and these organizations collectively support over 25,000 persons in addiction recovery who are living in over 2,500 certified recovery residences throughout the United States. **https://narronline.org**

National Association of Counties

The National Association of Counties (NACo) strengthens America's counties, including nearly 40,000 county elected officials and 3.6 million county employees. Founded in 1935, NACo unites county officials to: advocate for county government priorities in Federal policymaking; promote exemplary county policies and practices; nurture leadership skills and expand knowledge networks; optimize county and taxpayer resources and cost savings; and, enrich the public's understanding of county government. **https://www.naco.org**

National Association of Development Organizations

The National Association of Development Organizations (NADO) provides advocacy, education, research, and training for the nation's regional development organizations. The association and its members promote regional strategies, partnerships, and solutions to strengthen the economic competitiveness and quality of life across America's local communities. **https://www.nado.org/**

National Farmers Union

The National Farmers Union (NFU) was founded by ten family farmers in 1902 as the Farmers Educational Cooperative Union of America in Point, Texas. NFU represents family farmers, fishers, and ranchers across the country, with formally organized divisions in 33 States. The key to the success and credibility of the organization has been Farmers Union's grassroots structure in which policy positions are initiated locally. The policy process includes the presentation of resolutions by individuals, followed by possible adoption of the resolutions at the local, State, and national levels. Members and staff of the Farmers Union advocate these policy positions nationwide. NFU believes that good opportunities in production agriculture are the foundation of strong farm and ranch families, and that strong farm and ranch families are the basis for thriving rural communities. Vibrant rural communities, in turn, are vital to the health, security, and economic well being of our entire national economy. **https://nfu.org/**

National Rural Health Association

The National Rural Health Association (NRHA) is a national nonprofit membership organization with more than 21,000 members. The association's mission is to provide leadership on rural health issues through advocacy, communications, education, and research. NRHA membership consists of a diverse collection of individuals and organizations, all of whom share the common bond of an interest in rural health. **https://www.ruralhealthweb.org/**

National Sheriffs' Association

Chartered in 1940, the National Sheriffs' Association is a professional association dedicated to serving the Office of Sheriff and its affiliates through law enforcement education and training and through the provision of general law enforcement informational resources. NSA represents thousands of sheriffs, deputies and other law enforcement, public safety professionals, and concerned citizens nationwide. Through the years, NSA has provided programs for sheriffs, their deputies, chiefs of police, and others in the field of criminal justice to perform their jobs in the best possible manner and to better serve the people of their cities, counties, or jurisdictions. **https://www.sheriffs.org/**

NORC Walsh Center for Rural Health Analysis

The NORC Walsh Center for Rural Health Analysis was established in 1996 by Project HOPE and transferred to NORC at the University of Chicago in 2003 to study policy issues affecting healthcare and health status in rural America. The Walsh Center for Rural Health Analysis' mission is to conduct timely policy analysis, research, and evaluation that address the needs of policy makers, the healthcare workforce, and the public on issues that affect healthcare and public health in rural America. **https://www.ruralhealthresearch.org/centers/walsh**

NTCA–The Rural Broadband Association

NTCA–The Rural Broadband Association is the premier association representing nearly 850 independent, community-based telecommunications companies that are leading innovation in rural and small-town America. NTCA advocates on behalf of its members in the legislative and regulatory arenas, and it provides training and development; publications and industry events; and an array of employee benefit programs. **http://www.ntca.org/**

National Rural Transit Assistance Program

The National Rural Transit Assistance Program (RTAP) was created in 1987. The goals of the RTAP program are to promote the safe and effective delivery of public transportation services in rural areas and to facilitate more efficient use of public transportation resources. To meet those goals, the program focuses on the following objectives: Improving the quality of training and technical assistance resources available to the rural transit industry; encouraging and assisting State, local, and peer networks to address training and technical assistance needs; promoting the coordination of transportation services; and, building a national database of information about the rural transit industry. Since its inception, National RTAP has contributed to the achievement of these objectives at the national level by developing and distributing training materials, providing technical assistance, creating web-based applications, producing reports, publishing best practices, conducting research, and offering peer assistance for rural and tribal transit providers and State RTAP programs. **https://nationalrtap.org/**

The Pew Charitable Trusts

The Pew Charitable Trusts is an independent non-profit, non-governmental organization, founded in 1948. The mission is to: improve public policy by conducting rigorous analysis, linking diverse interests to pursue common cause and insisting on tangible results; inform the public by providing useful data that illuminate the issues and trends shaping our world; and, invigorate civic life by encouraging democratic participation and strong communities. **https://www.pewtrusts.org**

U.S. Department of Agriculture

The U.S. Department of Agriculture (USDA) is made up of 29 agencies and offices with nearly 100,000 employees who serve the American people at more than 4,500 locations across the country and abroad. USDA provides leadership on food, agriculture, natural resources, rural development, nutrition, and related issues based on public policy, the best available science, and effective management. USDA has a vision to provide economic opportunity through innovation, helping rural America to thrive; to promote agriculture production that better nourishes Americans while also helping feed others throughout the world; and to preserve our Nation's natural resources through conservation, restored forests, improved watersheds, and healthy private working lands. **https://www.usda.gov/**

U.S. Department of Agriculture, National Institute of Food and Agriculture

The USDA National Institute of Food and Agriculture (NIFA) was established by the Food Conservation and Energy Act of 2008 (the 2008 Farm Bill) to find innovative solutions to issues related to agriculture, food, the environment, and communities. One of four agencies that make up USDA's Research, Education, and Economics (REE) mission area, the agency is structured to direct Federal funding effectively to programs that address key national and global challenges. **https://nifa.usda.gov/**

U.S. Department of Agriculture, Center for Faith-Based and Neighborhood Partnerships

USDA's Center for Faith-Based and Neighborhood Partnerships reaches out and connects the government with non-profit organizations, both secular and faith-based, to help people in need. The Center helps communities connect with USDA priorities and programs to turn visions more effectively into reality. **https://www.usda.gov/our-agency/staff-offices/center-faith-based-and-neighborhood-partnerships**

Endnotes

1 National Institute on Drug Abuse (NIH) **https://www.drugabuse.gov/publications/media-guide/glossary**

2 Mack KA, Jones CM, Ballesteros MF. Illicit Drug Use, Illicit Drug Use Disorders, and Drug Overdose Deaths in Metropolitan and Nonmetropolitan Areas — United States. MMWR Surveill Summ 2017;66(No. SS-19):1–12. DOI: **http://dx.doi.org/10.15585/mmwr.ss6619a1**

3 NPR/Robert Wood Johnson Foundation/Harvard School of Public Health Publication: Public Opinion Poll Series; Harvard T.H. Chan School of Public Health Editor(s): NPR/RWJF/Harvard School of Public Health Life in Rural America Experiences and views from rural America on economic and health issues and life in rural communities. **https://www.rwjf.org/en/library/research/2018/10/life-in-rural-america.html**

4 Morning Consult on behalf of American Farm Bureau Federation and National Farmers Union. Conducted an online survey of 2,201 rural adults to determine General Attitudes and Perceptions of Addiction. October 26-29, 2017. **https://farmtownstrong.org/theopioidcrisis/**.

5 Center for Behavioral Health Statistics and Quality. (2015). 2014 National Survey on Drug Use and Health: Detailed Tables. Substance Abuse and Mental Health Services Administration, Rockville, MD.

6 Lipari, R.N. and Van Horn, S.L. *Trends in substance use disorders among adults aged 18 or older.* The CBHSQ Report: June 29, 2017. Center for Behavioral Health Statistics and Quality, Substance Abuse and Mental Health Services Administration, Rockville, MD. **https://www.samhsa.gov/data/sites/default/files/report_2790/ShortReport-2790.html**.

7 The Associated Press-NORC Center for Public Affairs Research (2018). *Americans Recognize the Growing Problem of Opioid Addiction* **http://www.apnorc.org/PDFs/Opioids%202018/APNORC_Opioids_Report_2018.pdf**.

8 NIDA. (2018, July 2). Media Guide. Retrieved from **https://www.drugabuse.gov/publications/media-guide** on 2019, June 17.

9 Centers for Disease Control and Prevention (CDC). Opioid Overdose. Data Overview. (2019). **https://www.cdc.gov/drugoverdose/data/index.html**

10 Mack K.A., Jones C.M., Ballesteros M.F. Illicit Drug Use, Illicit Drug Use Disorders, and Drug Overdose Deaths in Metropolitan and Nonmetropolitan Areas—United States. MMWR Surveill Summer 2017; 66 (No. SS-19):1-12. DOI: **http://dx.doi.org/10.15585/mmwr.ss6619a1**.

11 See Opioid Misuse Community Assessment Tool www.opioidmisusetool.norc.org

12 Mercer County Comprehensive Plan, August 2018 **http://www.mercercountywv.org/images/2018.06.28-Final-Plan.pdf**

13 CDC/NCHS, National Vital Statistics System, Mortality. CDC WONDER, Atlanta, GA: US Department of Health and Human Services, CDC; 2018. **https://wonder.cdc.gov**

14 Rural America at a Glance, 2018 Edition (November 1, 2018). USDA-ERS Economic Information Bulletin No. 200. Available at: **https://www.ers.usda.gov/webdocs/publications/90556/eib-200.pdf?v=5899.2**

15 U.S. Census Bureau. New Census Data Show Differences Between Urban and Rural Populations. United States Census Bureau **https://www.census.gov/newsroom/press-releases/2016/cb16-210.html. Published 2016**. Accessed November 1, 2019.

16 American Hospital Association, 2019 Rural Report: Challenges Facing Rural Communities and the Roadmap to Ensure Local Access to High Quality, Affordable Care. **https://www.aha.org/system/files/2019-02/rural-report-2019.pdf**.

17 B. Whitacre, P.S. Hartman, S. Boggs, V. Schott - 2008, Oklahoma State University, Evaluating the Economic Impact of Telemedicine in a Rural Community. **https://shareok.org/bitstream/handle/11244/49723/oksd_agec_1007_2013-01.pdf?sequence=1**; HRSA 2015 Policy Brief, Telehealth in Rural America available at **https://www.hrsa.gov/advisorycommittees/rural/publications/telehealthmarch2015.pdf**.

18 Appalachian Diseases of Despair, Prepared for the Appalachian Regional Commission, The Walsh Center for Rural Health Analysis, NORC at the University of Chicago (2017) available at **https://www.arc.gov/assets/research_reports/appalachiandiseasesofdespairaugust2017.pdf**.

19 Creating a Culture of Health in Appalachia: Disparities and Bright Spots, **https://healthinappalachia.org**/

20 Federal Rural Resources Guide **https://www.rd.usda.gov/files/RuralResourceGuide.pdf**.

21 Hanley, S.M., Ringwalt, C., Ennett, S.T., Vincus, A.A., Bowling, J.M., Haws, S.W., & Rohrbach, L.A. (2010). The prevalence of evidence-based substance use prevention curricula in the nation's elementary schools. *Journal of Drug Education*, 40(1), 51-60. doi:10.2190/DE.40.1.d; Griffin, K.W., & Botvin, G.J. (2010). Evidence-based interventions for preventing substance use disorders in adolescents. *Child and Adolescent Psychiatric Clinics of North America*, 19(3), 505–526. doi:10.1016/j.chc.2010.03.005.

22 Morning Consult on behalf of American Farm Bureau Federation and National Farmers Union. Conducted an online survey of 2,201 rural adults to determine General Attitudes and Perceptions of Addiction. October 26-29, 2017. **https://farmtownstrong.org/theopioidcrisis/**.

23 The Cecil G. Sheps Center for Health Services Research, available at **https://www.shepscenter.unc.edu/programs-projects/rural-health/rural-hospital-closures/**.

24 Siegel, Jessica, National Rural Health Association (Feb. 20, 2019) Rural Hospital Closures Rise to Ninety-Eight, available at **https://www.ruralhealthweb.org/blogs/ruralhealthvoices/february-2019/rural-hospital-closures-rise-to-ninety-seven**.

25 University of Michigan Behavioral Health Workforce Research Center. Characteristics of the Rural

Behavioral Health Workforce: A Survey of Medicaid/Medicare Reimbursed Providers. Ann Arbor, MI: UMSPH; 2018.

26 C. Holly A. Andrilla et al., "Geographic Distribution of Providers With a DEA Waiver to Prescribe Buprenorphine for the Treatment of Opioid Use Disorder: A 5-Year Update," *The Journal of Rural Health* (2018), **https://doi.org/10.1111/jrh.12307**.

27 Richard P. Mattick et. al., "Methadone Maintenance Therapy Versus No Opioid Replacement Therapy for Opioid Dependence," *Cochrane Database of Systematic Reviews* 3, no. CD002209 (2009), **http://www.ncbi.nlm.nih.gov/pubmed/19588333**; Sandra D. Comer et al., "Injectable, Sustained-Release Naltrexone for the Treatment of Opioid Dependence: A Randomized, Placebo-Controlled Trial," *Archives of General Psychiatry* 63, no. 2 (2006): 210-8, **http://archpsyc.jamanetwork.com/article.aspx?articleid=209312**; Paul J. Fudala et al., "Office-Based Treatment of Opiate Addiction With a Sublingual-Tablet Formulation of Buprenorphine and Naloxone," *New England Journal of Medicine* 349, no. 10 (2003): 949-58,

28 Mattick et al., "Methadone Maintenance"; Comer et al., "Injectable, Sustained-Release Naltrexone"; Fudala et al., "Office-Based Treatment"; Richard P. Mattick et al., "Buprenorphine Maintenance Versus Placebo or Methadone Maintenance for Opioid Dependence," Cochrane Database of Systematic Reviews 2, no. CD002207 (2014), **http://dx.doi.org/10.1002/14651858.CD002207.pub4**.

29 American Society of Addiction Medicine, "The ASAM National Practice Guideline."

30 John A. Gale, Anush Y. Hansen, and Martha Elbaum Williamson, "Rural Opioid Prevention and Treatment Strategies: The Experience in Four States," Maine Rural Health Research Center (2017), **https://muskie.usm.maine.edu/Publications/rural/WP62-Rural-Opioid-Prevention-Treatment-Strategies.pdf**.

31 Andrew Rosenblum et al., "Distance Traveled and Cross-State Commuting to Opioid Treatment Programs in the United States," *Journal of Environmental and Public Health* no. 948789 (2011), **https://doi.org/10.1155/2011/948789**.

32 C. Holly A. Andrilla et al., "Geographic Distribution of Providers With a DEA Waiver to Prescribe Buprenorphine for the Treatment of Opioid Use Disorder: A 5-Year Update," *The Journal of Rural Health* (2018), **https://doi.org/10.1111/jrh.12307**.

33 C. Holly A. Andrilla, Cynthia Coulthard, and Eric H. Larson, "Barriers Rural Physicians Face Prescribing Buprenorphine for Opioid Use Disorder," *Annals of Family Medicine* 15, no. 4 (2017): 359-62, **http://dx.doi.org/10.1370/afm.2099**.

34 Mary Bond Edmond, Lydia Aletraris, and Paul M. Roman, "Rural Substance Use Treatment Centers in the United States: An Assessment of Treatment Quality by Location," *American Journal of Drug and Alcohol Abuse* 41, no. 5 (2015): 449-57, **http://dx.doi.org/10.3109/00952990.2015.1059842**.

35 P. Todd Korthuis et al., "Primary Care-Based Models for the Treatment of Opioid Use Disorder: A Scoping Review," *Annals of Internal Medicine* 166, no. 4 (2017): 268-78, **http://dx.doi.org/10.7326/M16-2149**.

Intentionally Left Blank

Intentionally Left Blank

Inside Back Cover
