# Supplementary material for: Rural Community Toolbox to Help Battle Opioid Epidemic
Source: J Appalach Health. 2020 Sep 1;2(4):1–3. doi: 10.13023/jah.0204.01 (PMC9150490; doi:10.13023/jah.0204.01)
Supplement: Supplementary file 2 [file 2.4.1CareyAdditionalFile#2.pdf]

Office of National Drug Control Policy

Rural Community Toolbox Fact Sheet

- **Drugs in rural America**: Our country is in the midst of an unprecedented drug crisis. In 2018, nearly 68,000 Americans died due to a drug overdose. While that was the first decline in the number of drug overdose deaths in 30 years, this is still almost 200 people each day.

  The addiction crisis knows no economic or geographic boundary and has impacted small towns and rural places from Alaska to Maine. A 2017 survey conducted by the American Farm Bureau Federation and the National Farmers Union found that nearly 50 percent of rural adults, and 74 percent of farmers, have been directly impacted by opioid misuse.

- **Needs of rural communities**: Rural areas lack many of the resources needed to keep people healthy and free from addiction including critical assets like treatment centers, mental and behavioral health counselors, support groups, and transportation. The most effective solutions to filling these gaps often begin at a local level, not in Washington, D.C. To take action and create real change, however, local leaders need tools and the support of state and Federal partners.

- **Rural Community Toolbox**: As a committed partner to local leaders in combatting the addiction crisis in rural America, the Office of National Drug Control Policy (ONDCP) has built a Rural Community Toolbox, found at www.ruralcommunitytoolbox.org. The Rural Community Toolbox is a clearinghouse for funding, technical assistance, and other information from 16 Federal departments and agencies to support local action in rural America.

  Major departments and agencies featured in the Rural Community Toolbox include:

    - U.S. Department of Agriculture
    - U.S. Department of Commerce
    - U.S. Department of Education
    - U.S. Department of Health & Human Services
    - U.S. Department of Housing and Urban Development
    - U.S. Department of Justice
    - U.S. Department of Labor
    - U.S. Department of Transportation

- U.S. Department of Veterans Affairs
- Small Business Administration
- Environmental Protection Agency
- Appalachian Regional Commission
- Corporation for National and Community Service
- Federal Communications Commission
- National Rural Transit Assistance Program

In addition to funding and technical assistance, the Rural Community Toolbox includes a library of current informational resources from Federal departments and agencies on 46 key topics related to drug addiction in rural America, such as adverse childhood experiences; American Indians, Alaska Natives, and Native Hawaiian populations; economic development; healthcare workforce; housing and homelessness; infrastructure; medication-assisted treatment (MAT); recovery; suicide and suicide prevention; and transportation.

- **Background**: In late 2018, ONDCP/USDA released a Federal Rural Resources Guide, which is a comprehensive listing of Federal programs that can be used to address opioid misuse in rural communities. This product was developed by the ONDCP Federal Rural Interagency Working Group on Substance Use Disorder and became the foundation of the Rural Community Toolbox, which includes updated funding opportunities and an interactive platform.

- **Comprehensive resource**: Built to be a comprehensive resource for rural communities, the Rural Community Toolbox will also house the Community Assessment Tool and Rural Community Action Guide to help rural leaders with data, background information, and recommended action steps to address addiction in their community.

  - The Community Assessment Tool gives county-specific data about overdose deaths and factors that may make a community more vulnerable to addiction, such as unemployment rates and education levels. In June 2020, the Rural Interagency Working Group added new data sets to the tool including broadband availability, transportation, treatment facilities, healthcare professional shortage areas, economic development districts and persistent poverty counties. The tool was also updated to include a rural prosperity index to help rural leaders think about community resiliency.

  - The Rural Community Action Guide provides background information and recommended action steps from rural stakeholder partners on 15 different topics related to addiction – ranging from stigma to recovery housing and broadband access.
